# Characterization of *Ganoderma pseudoferreum* mitogenome revealed a remarkable evolution in genome size and composition of protein-coding genes

**DOI:** 10.3389/fpls.2025.1532782

**Published:** 2025-08-20

**Authors:** Jinpeng Lu, Chunxiu Qin, Siyan Huo, Huanwei Wang, Justice Norvienyeku, Weiguo Miao, Wenbo Liu

**Affiliations:** ^1^ Key Laboratory of Green Prevention and Control of Tropical Plant Diseases and Pests, Ministry of Education, School of Tropical Agriculture and Forestry, Hainan University, Haikou, China; ^2^ Danzhou Invasive Species Observation and Research Station of Hainan Province, Hainan University, Danzhou, China

**Keywords:** Ganoderma, mitogenome, tRNA, speciation, phylogenomics

## Abstract

Red root disease in rubber trees, caused by *Ganoderma pseudoferreum*, is a prevalent and severe soil-borne disease in rubber tree cultivation areas. The pathogen exhibits complex infections, with multiple transmission pathways, making the disease highly concealed and difficult to diagnose in its early stages. As a result, prevention and control are challenging, posing a serious threat to rubber production. Currently, the relevant information, evolutionary trajectory, and sequence divergence of the mitochondrial genome of *G. pseudoferreum* remain unknown. Here, we assembled the complete mitochondrial genome of *G. pseudoferreum*,which is 40, 719 bp long and contains 14 protein-coding genes (PCGs), genes encoding small and large ribosomal subunits, 22 mitochondrial-encoded tRNAs, and four hypothetical proteins. The genomic content and characteristics, along with IPS mapping analysis and phylogenetic analysis, reveal a significant similarity between *G. pseudoferreum* and *G. lingzhi.* The results of RNA editing site analysis, codon usage bias and evolutionary pressure analysis reveal that during environmental adaptation, species of Ganoderma may alter certain key PCGs to adopt distinct evolutionary trajectories, differentiating themselves from other fungi in Basidiomycota, while leaving evolutionary traces. Our study provides new insights into the evolutionary direction and pattern of *G. pseudoferreum* and *Ganoderma* by exploring the evolutionary trajectory of mitochondrial genomes of *G*. *pseudoferreum* and Ganoderma.

## 1 Introduction

Rubber tree (*Hevea brasiliensis* Muell. Arg.), an important economic crop in tropical regions, occupies a major economic position in tropical countries. (Wastie, 1975; Cai et al., 2013) Because it produces natural rubber (cis-1,4-polyisoprene), which is a crucial industrial raw material, the rubber tree has become an important component of the tropical agricultural economy. (Venkatachalam et al., 2007; Liang et al., 2023) Among the many diseases affecting the growth of the rubber tree, red root disease caused by *Ganoderma pseudoferreum* is one of the most serious diseases. Due to the formation of a pathogen-pest-soil pollution complex infection, the fungal mycelial network is difficult to eliminate. With diverse transmission pathways, the disease remains highly concealed, making early diagnosis challenging and resulting in difficult prevention and control. Especially among the seven major diseases causing root disease in rubber trees, it is the most serious root disease (Meng et al., 2024).

*Ganoderma* belongs to the Basidiomycota family Polyporaceae, which is widely distributed globally with diverse species and similar morphological characteristics. (Kües et al., 2015; Kwon et al., 2016; Jargalmaa et al., 2017) Owing to the antioxidant, antitumor, and antibacterial activity characteristics of *Ganoderma*, (Chan et al., 2015; Chien et al., 2015) as well as its rich and diverse bioactive components, (Baby et al., 2015; Cör et al., 2018) *Ganoderma* has been widely cultivated in the health and pharmaceutical industries as a nutritional medicine. (Kuok et al., 2013) However, the highly similar characteristics of *Ganoderma* spp. make classification and identification difficult. (Wang et al., 2012; Li et al., 2022) In recent years, owing to the unique genetic characteristics of the mitochondria, the mitochondrial genome has become a useful tool for studying the phylogeny and evolutionary trends of fungi. (Cameron, 2014; Yuan et al., 2015; Li et al., 2019b; Ji et al., 2023) There are numerous reports that *Ganoderma* infects plants and causes harmful diseases, such as plant pathogens. (Francis et al., 2014; Hidayati et al., 2014; Page et al., 2018; Zhang et al., 2018) However, research on the complete mitochondrial genome of *Ganoderma* was either still in its infancy. (Li et al., 2022) Among these studies, studies focusing on the mitochondrial genome characteristics and phylogenetic analyses of plant pathogenic species within the *Ganoderma* genus remain scarce.

Mitochondria are indispensable organelles in fungi that act as efficient energy-conversion platforms and vital hubs for life processes. (Osiewacz, 2002; Patkar et al., 2012; Kouvelis and Hausner, 2022; Ji et al., 2023) Mitochondria play an important role in providing ATP, participating in ion balance, and cell apoptosis. (McBride et al., 2006) The mitochondrial genome is considered the second largest genome after the nuclear genome. (Henze and Martin, 2003; Cieslak et al., 2010; Li et al., 2022) It contains useful molecular information that can be used to infer the evolutionary relationships between fungi within the same genus or species and between different fungal taxa. (Ballard and Whitlock, 2004; Slack et al., 2007) In the current mitochondrial genome research, a small portion has been occupied by studies on fungal mitochondrial genomes. The rarity of research on complete fungal mitochondrial genomes has been attributed to the diversity of fungal genetics and structural differences. (Barr et al., 2005; Freel et al., 2015; Li et al., 2022) In the NCBI database (Date of Visit: December 25, 2023), fewer than 490 complete mitochondrial genomes of fungal basidiomycetes were recorded. Compared to the number of complete mitochondrial genomes of animals (5,403) and plants (1,547), the number of fungal genomes is minuscule. The basic structure of the fungal mitochondria includes 14 protein-coding genes (PCGs) involved in the electron transport chain and oxidative phosphorylation. These are composed of Complex I (*nad1*-*nad6*), Complex III (*cob*), Complex IV (*cox1*, *cox2*, *cox3*), and Complex V (*atp6, atp8*, and *atp9*) (Li et al., 2018, 2019a) In addition, the mitochondrial genome contains two translation-related ribosomal RNAs (*rnl* and *rns*) and a set of approximately 20–36transfer tRNAs. (Kang et al., 2017; Li et al., 2021) Some fungi also possess a ribosomal small subunit (*rps3*). (Zardoya, 2020) In terms of the composition and gene structure of the fungal mitochondrial genome, such as intron feature differences, degree of gene displacement, repeat sequences, non-coding genes, and basic genome features, significant differences have been observed between genomes within the same genus.

Therefore, these differences can be used to evaluate the degree of evolution and evolutionary trends All mitochondrial genomes of *Ganoderma* published so far are derived from saprophytic species (Li et al., 2019b, 2022) The characteristics of the mitochondrial genome of hemibiotrophic plant pathogenic fungi have not yet been reported. It remains unknown whether differences in nutritional modes also cause differences in mitochondrial genomes. In the present study, we sequenced and assembled the mitochondria of *G. pseudoferreum*, a pathogen that causes red root disease in rubber trees. Based on the published mitochondrial genomes of the Ganoderma, the degree of differences in mitochondrial genomes at the interspecific level was compared.

Furthermore, based on some of the published mitochondrial genomes of Basidiomycota, differences in mitochondrial genomes at the intergeneric level were analyzed, including phylogenetic status, mitochondrial genome composition, codon usage bias, and gene selection pressure. In summary, this comprehensive study revealed characteristic differences in the mitochondrial genome of *G. pseudoferreum* and assessed the degree of evolution and evolutionary trend of *G. pseudoferreum*. This provides new knowledge for the study of the mitochondrial genome of Ganoderma, which has helped us understand the evolutionary patterns of fungi in *Ganoderma* and has guiding significance for the genetic classification of *Ganoderma*.

## 2 Methods

### 2.1 Sampling, DNA extraction and sequencing

*Ganoderma pseudoferreum* strain HNDZHG02 was isolated from the Danzhou Rubber (Hevea brasiliensis Reyan 7-33-97) Forest, Hainan Province, China. Diseased roots were surface-sterilized, sectioned, and incubated under high-humidity conditions. After white mycelia emerged from the cut surfaces, segments were transferred to Potato Dextrose Agar (PDA) slants for storage at room temperature (25 ± 1°C). Mycelial plugs were then cultured on PDA plates for 14 days to assess morphological characteristics. Non-infected roots were inoculated and incubated under identical high-humidity conditions for comparative morphological observation. The pathogen was then isolated and purified. The pathogen was cultured on PDA culture medium plate and preserved at the Key Laboratory of Green Prevention and Control of Tropical Plant Diseases and Pests. Genomic DNA was extracted using CTAB commercial kits (Guangzhou Chemical Reagent Factory Co., LTD),according to the manufacturer’s instructions. DNA concentration was measured by Qubit^®^ DNA Assay Kit in Qubit 3.0 Flurometer (Invitrogen, Carlsbad, CA, USA). Sequencing library was generated using NEB Next Ultra DNA Library Prep Kit for Illumina (New England Biolabs, Ipswich, MA, USA) following manufacturer’s recommendations and index codes were in addition to each sample. Clustering of the index-coded samples was performed on a cBot Cluster Generation System using an Illumina PE Cluster Kit (Illumina, San Diego, CA, USA) according to the manufacturer’s instructions. After cluster generation, the DNA libraries were sequenced on an Illumina platform and 150 bp paired-end reads were generated. Genome DNA sequencing was completed by Huitong Biotechnology Co., Ltd. (Shenzhen, China). A series of quality control steps was performed to process the raw data. We used Fastp (version 0.19.7) to perform basic statistics on the quality of the raw reads as follows: Paired reads were discarded if they contained adapter contamination, had >10% uncertain bases, or had >50% low-quality (Phred <5) bases in either read.

### 2.2 Assembly of mitochondrial genome

To assemble the mitochondrial (mt) genome of *G. pseudoferreum* strain HNDZHG02, the preliminary assembly of quality-controlled scaffolds was performed using SPAdes software. The k-mer settings were optimized using VelvetOptimiser, and the assembly process was performed with k-mer values set to 93, 95, 97, 103, 105, 107, and 115. After assembly, the final results were integrated. The final assembly was obtained by integrating the best results. Subsequently, published mitochondrial genome data and protein-coding sequences were used as references for comparison using BLASTn and Exonerate, with a nucleotide alignment threshold of 1e−10 and a protein similarity cutoff of 70%. Scaffolds with matching genes were selected, and the coverage of the assembly was sorted to remove fragments that were not part of the target genome. To extend and merge these candidate fragments, PRICE and MITObim were employed with 50 iterations to reduce scaffold fragmentation. For the iterative assembly, bowtie2 was used to map the original sequencing reads, and the matched paired reads were selected and reassembled using SPAdes. Finally, the assembly path was evaluated, and the circular mitochondrial genome was extracted.

### 2.3 Annotation of mitochondrial genome

The assembled mt genomes were initially annotated with the MFannot webserver (Zardoya, 2020; Lang et al., 2023; Valach et al., n.d) using genetic code 4 (Mold, Protozoan, and Coelenterate Mt Code). The initial genes predicted by MITOS were deduplicated and the start and stop codon positions of the genes were manually corrected to obtain a highly accurate set of conserved genes. (Bernt et al., 2013) Using NCBI-BLAST, homologous genes were aligned to examine conserved N-terminal and C-terminal regions. This enabled correction of start and stop codon accuracy and completeness by adjusting initiation and termination sites. These adjustments ensure no premature stop codons are introduced while preventing truncation of conserved domains. The coding proteins, tRNA, and rRNA of the mt genomes were predicted using MITOS software (Donath et al., n.d), the secondary structure of tRNA genes was predicted using tRNAscan-SE v.2.0 (http://lowelab.ucsc.edu/tRNAscan-SE/), and the secondary structure of tRNA was visualized using VARNA software. Introns were classified as group I or II using RNAweasel. (Lang et al., 2007; Ji et al., 2023) Intronic Open Reading Frame (ORF) were detected using ORFfinder v0.4.3. (Sayers et al., n.d; Wheeler et al., n.d)Using a minimum ORF length of 150 bp and genetic code of four. Conserved functional domains and superfamilies were identified by querying ORF sequences (>150 bp) with e-values <1e-3 within intron ORFs in the NCBI Conserved Domain Database (CDD), (Marchler-Bauer et al., n.d) and ORFs encoding homing endonucleases (HEs) of the LAGLIDADG or GIY-YIG family or reverse transcriptases (RTs) were identified and classified according to their conserved domains. Short exact repeats were identified using REPuter, and tandem repeats were identified using Tandem Repeat Finder v4.09. (Benson, n.d; Kurtz and Schleiermacher, n.d) A circular illustration of the mitochondrial genome was constructed using SnapGene v6.02 and CGview.

### 2.4 Indexes of intron position sets

To identify the insertion positions of introns, the coding regions of a set of homologous genes(%Identity>90%,e-value<1e-5)were aligned with MAFFT v7.520 (The L-INS-i strategy from the Iterative Refinement approach was used to align the main alignable regions, utilizing the Smith-Waterman algorithm). (https://mafft.cbrc.jp/alignment/server/) Using genetic code 4 and codon-based alignment was performed by integrating amino acid sequences and CDS sequences to determine intron insertion sites (Katoh et al., n.d). Introns in the same IPS are believed to be common among species (Wang et al., 2020).

### 2.5 Sequences analysis

The base composition was analyzed using SnapGene v6.02 and MEGA v11.08. Strand asymmetry were computed manually using the following formulas: GC-skew= (G-C)/(G + C) and AT-skew = (A-T)/(A + T). (Caspermeyer, 2016; Wang et al., 2020) DnaSP v6.10.01 (Rozas et al., 2017) was used to calculate the synonymoussubstitution rate (Ks), the non-synonymous substitution rate (Ka) and Ka/Ks values, using “Yang& Nielsen (2000) method”. Genome synteny of *G. pseudoferreum* mitogenome and other previously published Ganoderma mitogenomes was analyzed using TBtools v2.001. (Rozas et al., 2017) Deepred-mt (Edera et al., 2021) was used to predict RNA editing sites in 14 PCGs (*apt6, atp8, atp9, nad1, nad2, nad3, nad4, nad4L, nad5, nad6, cox1, cox2, cox3, cox3, cob*). The predicted results with probability values greater than or equal to 0.6.

### 2.6 Phylogenetic analysis

Mitogenomic sequences and annotations of filamentous fungi from 97 saprophytic and phytopathogenic fungi in Basidiomycetes were downloaded from the NCBI database, which contains definite species and numbers. The 14 PCGs (*apt6, atp8, atp9, nad1, nad2, nad3, nad4, nad4L, nad5, nad6, cox1, cox2, cox3, cox3, cob*) were aligned using MAFFT v7.505. (Katoh et al., n.d.) A phylogenetic tree was constructed using the maximum-likelihood method (ML) in MEGAv11.08 (Caspermeyer, 2016) with a bootstrap value of 1000. *Phytophthora infestans* was selected as the outgroup. The phylogenetic tree was visualized using iTOL v5 (Letunic and Bork, 2021) software.

### 2.7 Data availability

The complete mitogenome of *G. pseudoferreum* was submitted to the GenBank database under accession number PP778499.

## 3 Results and analysis

### 3.1 Structure and organization of the mitochondrial genomes

**Figure 1:**
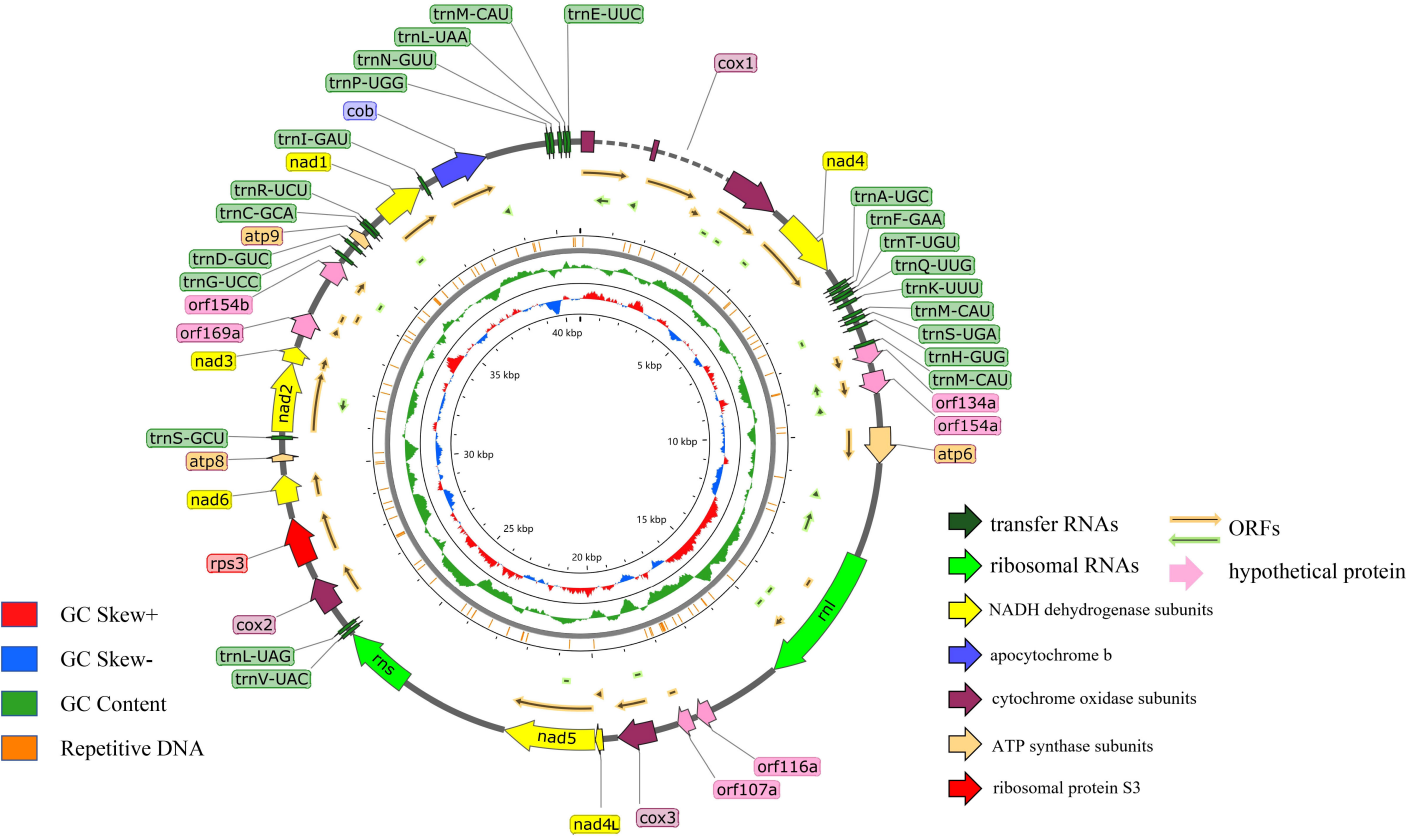
Circular illustration of the mitogenome of the *G. pseudoferreum*. Various genes are represented with different color arrows. The different color rings from inside to outside represent the GC-skew distribution, GC contents of non-overlapping sliding windows of 500 bp and repetitive DNA sequence regions in the mitogenomes.

The assembled *G. pseudoferreum* mitochondrial genome yielded a gapless circular DNA sequence with a total length of 40,719 bp. The entire mitochondrial genome consists of 14 standard protein-coding genes (PCGs), including NADH dehydrogenase subunits (*nad1, nad2, nad3, nad4, nad4L, nad5, nad6*), ATP synthase subunits (atp6, apt8, atp9), cytochrome oxidase subunits (*cox1, cox2* and *cox3*) and apocytochrome b (*cob*), and 2 ribosomal RNAs (*rnl* and *rns*). The complete mitochondrial genome of *G. pseudoferreum* transcribes 22 tRNAs, and 4 ORFs were predicted to encode hypothetical protein-coding genes. All coding genes in the *G. pseudoferreum* mitochondrial genome were transcribed from the majority strand (J-strand) (**Figure 1**).

The GC content of the *G. pseudoferreum* mitochondrial genome was 26.4%, with GC-skew and AT-skew values of 0.0378 and -0.0108, respectively. The repeated DNA fragments account for 5.28% of the mitochondrial genome (**Table 1**). *G. pseudoferreum* has only two introns, both of which are located in cox1 Compared to 12 closely related species, it had the lowest intron content, but the GC content of the introns was similar (**Supplementary Figure 1**). These two introns belong to Group I, and each carries an ORF encoding a homing endonuclease, both of which belong to the LAGLIDADG family (**Table 1**). Except cox1, the remaining genes contained no introns (**Table 2**).

**Table 1:** Assembly statistics of *G. pseudoferreum* mitogenome.

Genome features	*Ganoderma pseudoferreum*
Total size(bp)	40719
Over GC(%)	26.4
GC-skew (G − C)/(G + C)	0.0378888
AT-skew (A − T)/(A + T)	-0.01087
Repetitive DNA(%)	5.28
Genes	45
mt tRNA gene number	22
Introns	2
Group I introns	2
Group II introns	0
Intergenic regions(bp)	15389
Intronic regions size (bp)	2958
Coding region GC(%)	28.97
Intergenic regions GC (%)	37.79
Intronic regions GC (%)	27. 14
Number of ORFs (bp)	16878
Intronic ORFs	2
LAGLIDADG ORFs	2
GIY-YIG ORFs	0
Reverse transcriptase ORFs	0

**Table 2:** The overall features of the core mitochondrial genes of *G. pseudoferreum*.

Gene	Length (bp)	Intron number	Exonic region (bp)	Intronic region(bp)	Intronic region (%)	Start codon	Stop codon
atp6	774	0	774	0	0	ATG	TAA
atp8	159	0	159	0	0	ATG	TAA
atp9	222	0	222	0	0	ATG	TAA
nad1	1017	0	1017	0	0	ATG	TAA
nad2	1509	0	1509	0	0	ATG	TAG
nad3	360	0	360	0	0	ATG	TAA
nad4	1365	0	1365	0	0	ATG	TAA
nad4L	267	0	267	0	0	ATG	TAA
nad5	1983	0	1983	0	0	ATG	TAA
nad6	612	0	612	0	0	ATG	TAA
cox1	4545	2	1587	2958	65.08	ATG	TAA
cox2	756	0	756	0	0	ATG	TAA
cox3	810	0	810	0	0	ATG	TAA
cob	1158	0	1158	0	0	GTG	TAA
rps3	1017	0	1017	0	0	ATG	TAG
rnl	3069	0	3069	0	0	–	–
rns	1538	0	1538	0	0	–	–

### 3.2 Mitogenome conservation among the *Ganoderma* species

Compared with the 12 published closely related species of *Ganoderma*, namely *G. applanatum* (Wang et al., 2016a), *G. calidophilum*, (Wang et al., 2016a) *G. flexipes*, *G. leucocontextum*, (Wang et al., 2016a) *G. lingzhi*, *G. lucidum*, (Chen et al., 2012) *G. meredithae*, (Wang et al., 2016b) *G. multipileum*, *G. sichuanense*, (Li et al., 2019b) *G. sinense*, *G. subamboinense*, (Li et al., 2022) and *G. tsugae*, (Li et al., 2019b) the size of the mitochondrial genome of *G. pseudoferreum* and *G. lingzhi* was similar, at 40,719 bp and 49,234 bp respectively, both not exceeding 50,000 bp. The mitochondrial genome sizes of *G. applanatum, G. calidophilum, G. flexipes* were 119,803 bp, 124,588 bp, 107,774 bp, respectively, which were the three largest among the 13 closely associated species of *Ganoderma*. The mitochondrial genome sizes of the remaining 8 *Ganoderma* species ranged from 60,000 to 93,000 bp (**Supplementary Table 1**). The mitochondrial genomes of the 13 Ganoderma species had similar GC contents, varying from 25.0% to 27. 1%. More than half of the species have a GC content of approximately 26%(*G. pseudoferreum*, *G. applanatum*, *G. leucocontextum*, *G. lingzhi*, *G. lucidum*, *G. meredithae*, *G. sichuanense*, *G. sinense*, *G. subamboinense*, *G. tsugae*, **Supplementary Table 1**). The GC content of the coding regions in the mitochondrial genomes ranged from 26.45% to 28.97%. The GC content of the intronic regions ranged from 25.49% to 28.3%. The three GC contents exhibited similar overall trends among the 13 closely related species. However, in terms of the proportion of DNA repeat segments in the mitochondrial genome, *G. pseudoferreum* had the highest proportion (5.28%), whereas the remaining closely related species had proportions ranging from 1.68% to 3.62% (**Supplementary Figure 1**). Except for *G. leucocontextum*, which had a GC-skew of −0.0095, the GC-skew of the mitochondrial genomes of the entire Ganoderma ranged from 0.0377 to0.0656. The AT skew of the mitochondrial genomes of the entire Ganoderma ranged from−0.0109 to −0.0311. The proportion of intergenic regions ranged from 26.82% to 60. 10%, with most regions varying from 40% to 50%. The proportion of introns in *G. pseudoferreum* was 7.26%, which was significantly lower than that in the other 12 closely related species of *Ganoderma* (**Supplementary Table 1**).

**Figure 2:**
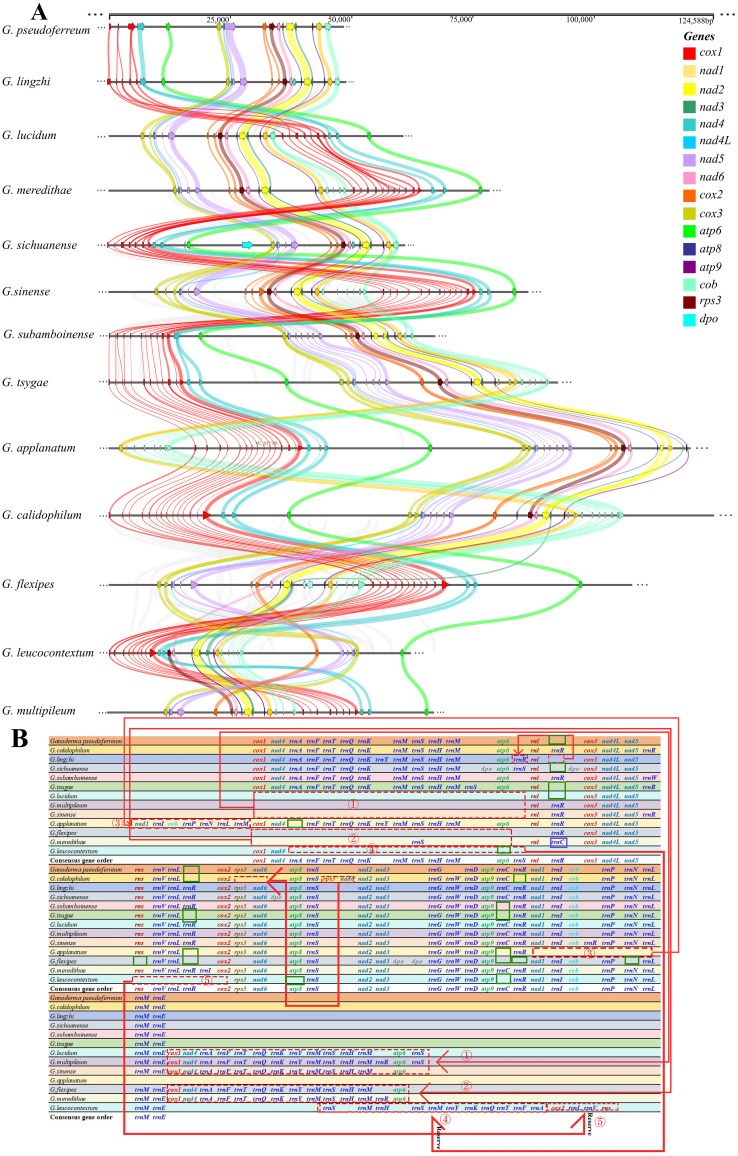
Comparison of mitochondrial genomes in 13 closely related Ganoderma species **(A)** Analysis of synteny among species within the *Ganoderma*. Different colored arrows represent 16 core coding genes, and right and left arrows represented positively and negatively transcribed genes, respectively. The bands between the intervals represent homologous regions among species. **(B)** Comparison of gene order among species within the *Ganoderma*. Different colored fonts represent different types of genes. Red dashed boxes represent gene order changes, blue boxes represent gene variations at this locus, and green boxes represent gene deletions at this locus.

A comparison of the mitochondrial genomes within the genus *Ganoderma* (**Figure 2A**) revealed that all 13 closely related species had 14 PCGs (*cox1, cox2, cox3, nad1, nad2, nad3, nad4, nad4L, nad5, nad6, atp6, atp8, atp9*). Except for *G. flexipes*, all other closely related species harbored *rps3*. Additionally, *G. flexipes, G. lingzhi*, and *G. sichuanense* have DNA polymerase(*dpo*). The results showed definite differences among closely related species, especially in the clustering of *cox1*, *nad4*, and *atp6* in the mitochondrial genomes, which underwent rearrangement.

However, the order of the three core PCGs, cox1, nad4, and atp6, was conserved in Ganoderma, 240 whereas the remaining PCGs(*cox3, nad4L, nad5, cox2, rps3, nad6, atp8, nad2, nad3, atp9, nad1, cob*) were conserved in terms of their loci and order. However, in *G. leucocontextum*, these core PCGs differed from those of the other 12 closely related species in terms of loci, order, and direction, indicating a rearrangement among the gene.

Further analysis of the gene rearrangement phenomena in 13 closely related species of the *Ganoderma* (**Figure 2B**), the results showed a cluster of genes (*cox1, nad4, trnA, trnF, trnT, trnQ, trnK, trnM. trnS, trnH, trnM, atp6*) underwent a shift within *G. lucidum, G. multipileum, G. sinense, G. flexipes*, and *G. meredithae*. A cluster of genes in *G. leucocontextum* underwent a flip-and-shift, and *atp8* and *atp9* were lost during gene rearrangement. Simultaneously, during gene rearrangement, *rns* is lost in *G. flexipes*. Compared with the aforementioned closely related species that lost core key coding protein genes, the remaining closely related species (*G. calidophilum*, *G. tsugae*, *G. applanatum* and *G.flexipes*) lost or expanded the number and type of tRNAs during the process of gene rearrangement or evolution. In contrast to these closely related species, the gene order of the mitochondrial genome of *G*. *pseudoferreum* was relatively conserved, with no large-scale gene displacement or deletion.

**Figure 3:**
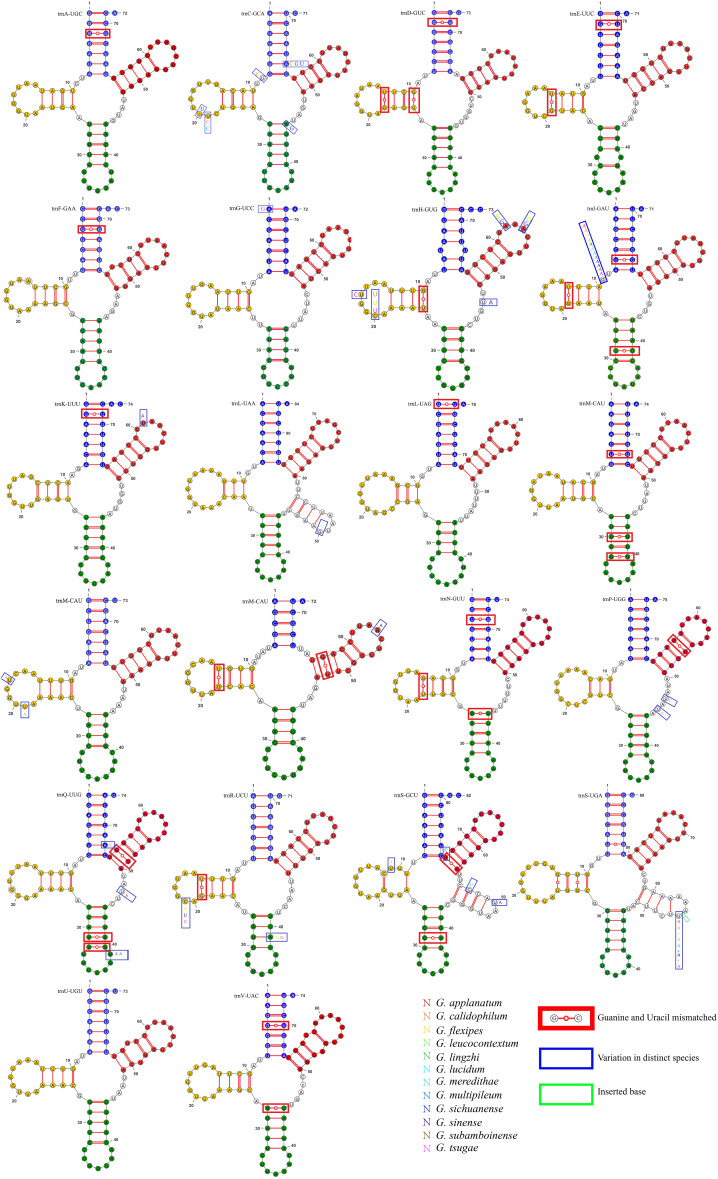
Variation in tRNA of *G*. *pseudoferreum* blue bases represented the acceptor stem. Yellow bases represented the Dihydrouridine stem-loop (DHU stem-loop). Green bases represented the Anticodon stem-loop structure. Red represented the TψC stem-loop structure. Base mismatches occurred in the red boxes. Variations at this locus in other closely related species occurred in the blue boxes, with different base colors representing different closelyrelated species. Base insertions at this locus in other closely related species occurred in the green boxes.

Upon visualizing the secondary structure of tRNA in 13 closely related species, we found that *G. applanatum, G. flexipes, G. leucocontextum, G. subamboinense*, and *G. tsugae* lack the mitochondrial tRNA-encoding cysteine (trnC-GCA). Among the published mitochondrial genomes of Basidiomycota in NCBI, *G. meredithae* uniquely possesses a tRNA (trnI-UAU) that encodes isoleucine. Compared to other closely related species, *G. subamboinense* possesses a unique tRNA (trnW-UCA) that encodes tryptophan. In contrast, *G. leucocontextum* lacks tRNA(trnR-UCG) which encodes arginine. The tRNA (trnM-CAU), encodes methionine in 13 closely related species of Ganoderma, had three copies. These results are generally consistent with those of published mitochondrial genomes. In addition to the above results, tRNA encoding arginine(trnR-UCG) in *G. sinense* and tRNA encoding arginine (trnR-UCU) in *G*. *multipileum* were analyzed in triplicate. Moreover, the tRNA encoding arginine (trnR-UCU) in *G. applanatum, G. meredithae*, and *G. subamboinense*, and the tRNA encoding serine (trnS-GCU) in G. lucidum, *G. multipileum*, and *G. sichuanense* were duplicated. All the remaining tRNAs were represented as single copies (**Supplementary Figure 2**). Upon observing the tRNA variations in *G. pseudoferreum*, it was found that 16 tRNAs (72.72%) exhibited mismatches, 13 tRNAs (59.09%) showed site mutations, and only one tRNA had a single-base insertion event on the Dihydrouridine loop (DHU loop) and DHU stem, anticodon stem, TψC loop, and TψC stem. However, no significant variations were observed in the anticodon loop or overall function (**Figure 3**).

### 3.3 Intron among the Ganoderma species

**Figure 4:**
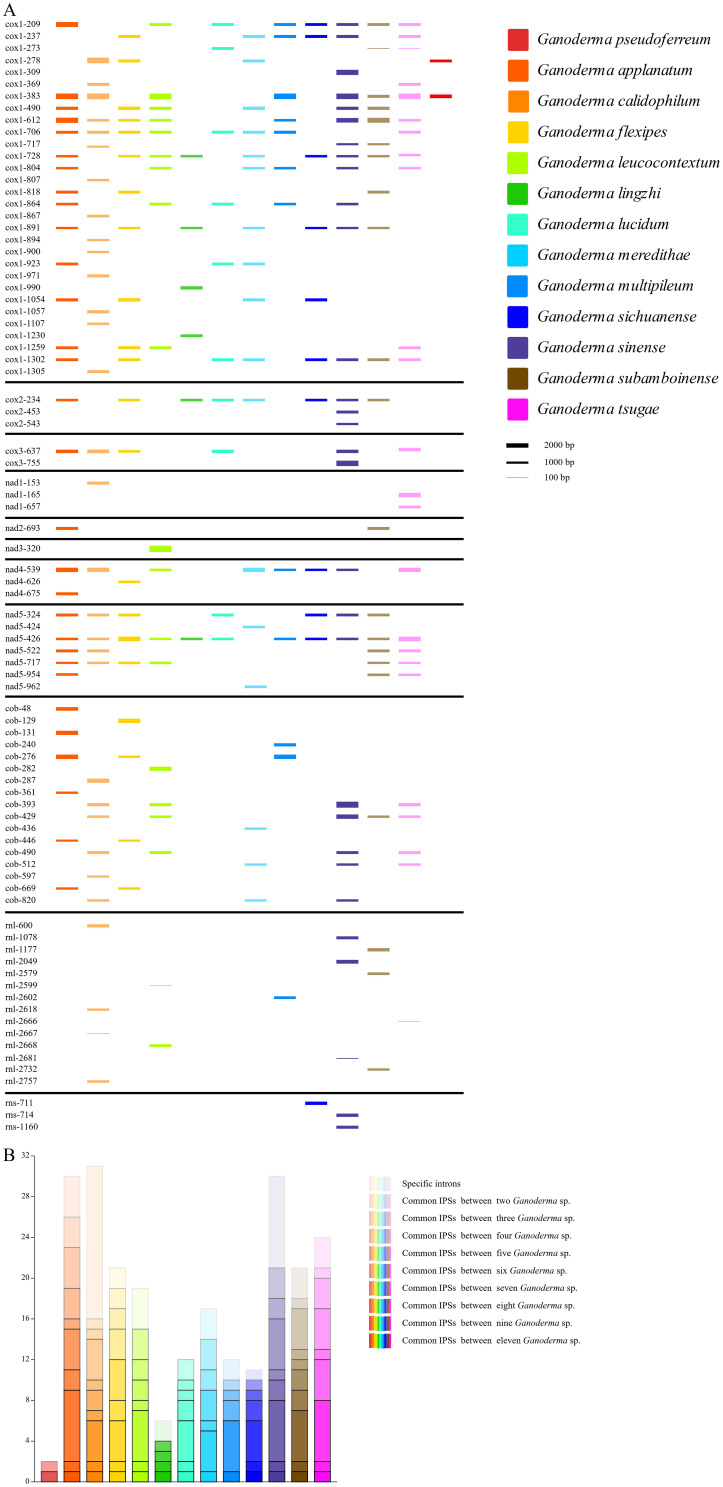
Intron position sets (IPSs) in mitogenomes of the 13 closely related species of the *Ganoderma* genus. **(A)** Dynamic bar chart of intron positions among closely related species. Different colors represent different closely related species. The intron insertion sites are determined by the mitochondrial genome. The height of each intron insertion barcode is determined by the length of the intron. **(B)** Bar chart of common intron insertion sites among closely related species. The color of the closely related species uses the color legend in Figure **(A)**. Different color transparencies represent the number of common IPSs, with the transition from light to dark representing an increase in the number of common IPSs.

Significant differences were observed in the content, insertion sites, and quantity, density, and size of introns in the mitochondrial genomes of 13 closely related species of *Ganoderma*. *G. applanatum, G. calidophilum* and *G. sinense* had the highest number of introns (30, 31, and 30). Moreover, *G. calidophilum* and *G. sinense* have higher numbers of introns with 16 and 9 specific introns, respectively. Subsequently, 21, 19, 17, 21, 24 number of introns were identified in *G. flexipes, G. leucocontextum, G.meredithae, G. subamboinense*, and *G. tsugae*, respectively. In contrast, *G. lucidum*, *G. multipileum, and G. sichuanense* had fewer introns, with 11, 12, and 11 introns identified respectively. *G. pseudoferreum* and *G. lingzhi* had the fewest introns among closely related species, with only two and six introns identified, respectively. (**Figure 4A**) On the other hand, in terms of intron density, *G. pseudoferreum* had the lowest density distribution at only 0.049 introns per kb. The density distribution of the other 12 closely related species varied from 0. 122 to 0.347 introns/kb, with most ranging from 0. 18 and 0.26 (**Supplementary Table 1**).

An intron position set (IPSs) was constructed among closely related species of the genus Ganoderma, characterized by the size and insertion sites of the introns. There were 84 IPSs identified in the genus Ganoderma (**Figure 4B**). Compared to the other 12 closely related species, *G. pseudoferreum* showed significant differences. Only two introns (cox1-278, cox1-383) in cox1.The cox1–278 position is not conserved and exists only in *G. calidophilum, G. flexipes*, and *G.meredithae*. The position cox1–383 was relatively conserved, with eight closely related species all having contained cox1-383. Frequent intron-loss events may have occurred in cox1. Overall, cox1in Ganoderma had the most intron insertions, and the majority of IPSs were relatively conserved, which is consistent with the published mitochondrial genome. In addition, the introns inserted in the cob were generally not conserved, with species-specific introns of closely related species primarily found in areas of cob in the IPSs. Relatively conserved IPSs in cox2, nad4, and nad5.Notably, the position nad5–426 was extremely conserved, being a conventional IPS of 11 closely related species (**Figure 4A**). None of the closely related species in the Ganoderma do not contain introns in nad4L, nad6, or ATP synthase subunits (atp6, atp8, atp9). Among the non-coding genes *rnl* and *rns*, IPSs were generally absent in closely related species, with only a few species possessing introns, all of which were species-specific IPSs (**Figure 4A**).

### 3.4 Phylogenetic analysis among Basidiomycota

The mitochondrial genomes of filamentous Basidiomycota were extracted from the fungal mitochondrial genomes available in the NCBI database. Fungal species labeled with “sp.”, i.e., unspecified species, were excluded. Pathogenic fungi from humans, animals, and insects were also omitted. Only saprophytic and plant pathogenic fungi were incorporated into the construction of the phylogenetic tree. In total, 96 fungal species, representing 31 genera, were selected (**Supplementary Table 2**). The maximum likelihood (ML) method was used to construct the phylogenetic tree. A Bootstrap value of 1000 was selected and Phytophthora infestans was used as the outgroup. A phylogenetic tree was constructed by selecting 14 core protein-coding genes (PCGs) from the mitochondrial genome in the following specific order (*atp6, atp8, atp9, cox1, cox2, cox3, cob, nad1, nad2, nad3, nad4, nad4L, nad5, nad6*). In the ML method, partitioning substitution models corresponding to the multigene alignment partitions were applied (**Supplementary Table 3**).

**Figure 5:**
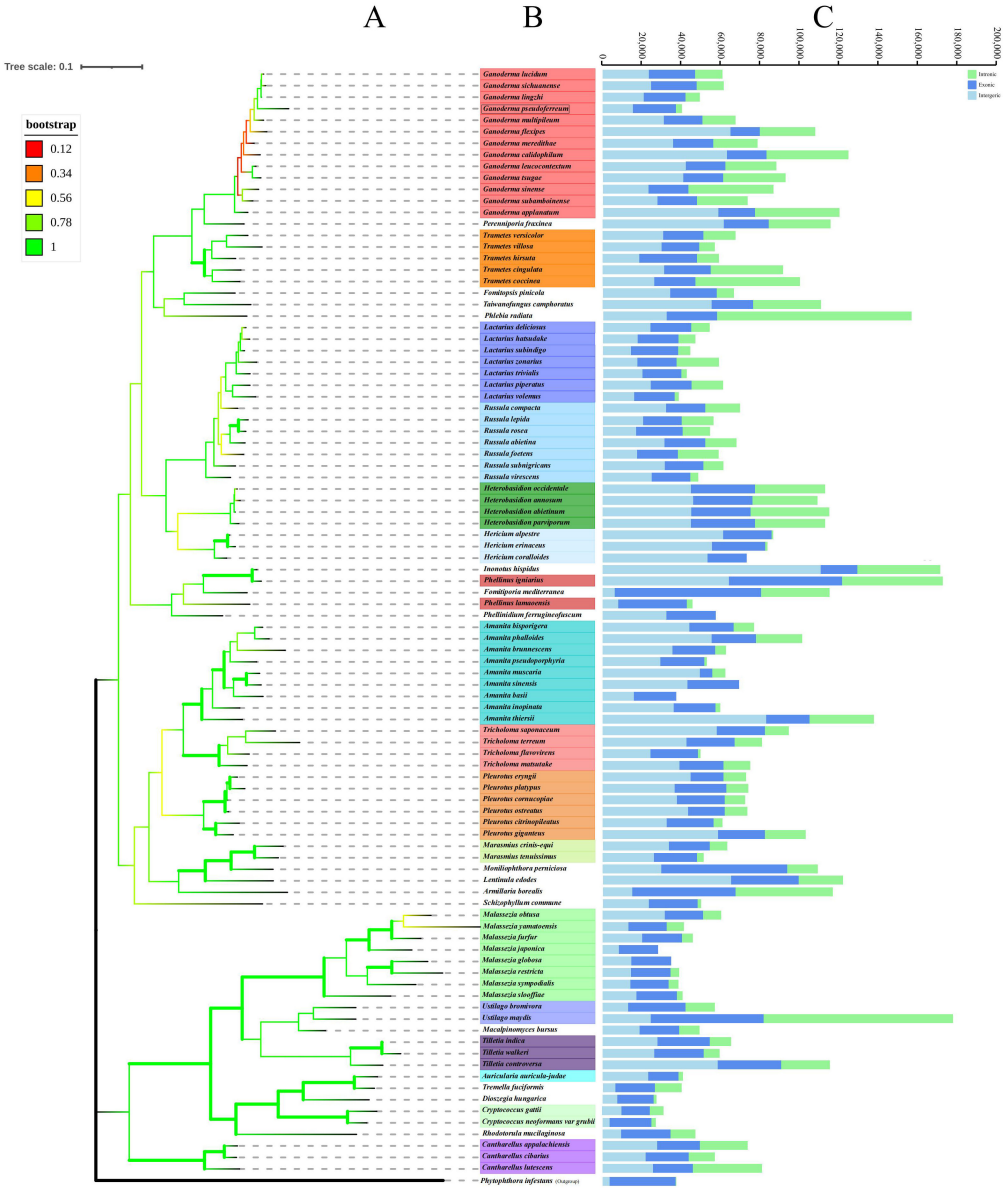
Phylogenetic trees of 97 species of filamentous PCGs of basidiomycetes were constructed based on maximum likelihood method (ML). **(A)** Tree topology and species list showing the branching of lineages. **(B)** The bootstrap values of tree nodes were color coded. **(C)** The components and size of the corresponding species genome.

Consequently, a Basidiomycota phylogenetic tree was constructed to determine the evolutionary relationship between Ganoderma genus and *G. pseudoferreum* (**Figure 5A**). The phylogenetic tree constructed with 97 species of Basidiomycota confirmed the diversity classification results and demonstrated good branching. The phylogenetic tree diagram indicated that *G. pseudoferreum* has a definite genetic relationship with *G. lucidum, G. sichuanense*, and *G.lingzhi*. In particular, *G. lingzhi* showed significant similarity to *G. pseudoferreum* in terms of the mitochondrial genome length and the length of each component. Species with close genetic relationships generally exhibit consistent or similar characteristics in terms of the size, composition, GC content, and gene conservation within the mitochondrial genome. However, this phenomenon was not observed within the same genus (**Figure 5**). The mitochondrial genomes of these 97 fungi exhibited substantial size variations, extending from 177,540 bp in *Ustilago bromivora* to 24,874 bp in *Cryptococcus neoformans* var. *grubii*. Moreover, considerable differences were observed in the sizes of the exon, intron, and intergenic regions. The exon region ranged from 74,214 bp in *Fomitiporia mediterranea* (64.51% of the mitochondrial genome) to6,330 bp in A. muscaria (10. 17% of the mitochondrial genome). The intron region ranged from98,593 bp in *Phlebia radiata* (accounting for 63.06% of the mitochondrial genome) to 0 bp. The intergenic region ranged from 110,571 bp in *Inonotus hispidus* (accounting for 64.64% of the mitochondrial genome) to 3,592 bp in *C. neoformans* var. *grubii* (accounting for 14.44% of the mitochondrial genome) (**Figure 5**). However, the size distributions of exon and intron regions were relatively concentrated. The mitochondrial genome exon region lengths of 90 species of Basidiomycota (accounting for 93.75% of all species) were significantly concentrated between15,000 and 35,000 bp. The lengths of the mitochondrial genome intron regions of 67 species, accounting for 69.79% of all species, were primarily between 0 and 20,000 base pairs (**Supplementary Figure 3A**). The proportion of each region’s length to its respective mitochondrial genome was similarly distributed, with a broad distribution across all regions (**Supplementary Figure 3B**). This indicates minimal evolutionary divergence in Basidiomycota mitochondrial genome functional regions of Basidiomycota but substantial variation in the intergenic and intron regions. This further suggests that the broad distribution of coding region lengths is due to the extensive distribution of intron region lengths despite the concentrated distribution of exon region lengths.

Based on the phylogenetic relationships among closely related species of the Ganoderma, further comparisons of mitochondrial genomes were made to calculate the divergence times of each closely related species using MCMCtree (rgene_gamma = 2 6, indicating the substitution rate was 0.33 substitutions per 100 Myr.). Interestingly, the branches of closely related species of the *Ganoderma* in terms of divergence time showed significant differences from the branches of closely related species of the Ganoderma in the phylogenetic tree based on mitochondrial genomes. However, in the time-evolutionary tree, *G. pseudoferreum, G. lucidum, G. sichuanense* and *G. lingzhi* clustered together, which is consistent with the clustering branches in the phylogenetic tree. From the results of divergence, the common ancestor of *G. pseudoferreum, G.lucidum, G. sichuanense*, and *G. lingzhi* diverged separately at 165.21 Mya (million years ago) and at 89.60 Mya, *G. pseudoferreum* diverged separately from this lineage, while the common ancestor of *G. lucidum, G. sichuanense*, and *G. lingzhi*, after a long evolutionary process of about 88 million years, completed divergence at 0.68~ 1.36 Mya (**Supplementary Figure 4**). The results of the time-evolutionary tree also better explain that *G. pseudoferreum* is closer to *G. lucidum, G.0sichuanense*, and *G. lingzhi* in terms of phylogenetic relationships.

### 3.5 Intergenus cluster analysis of mitogenomic composition

**Figure 6:**
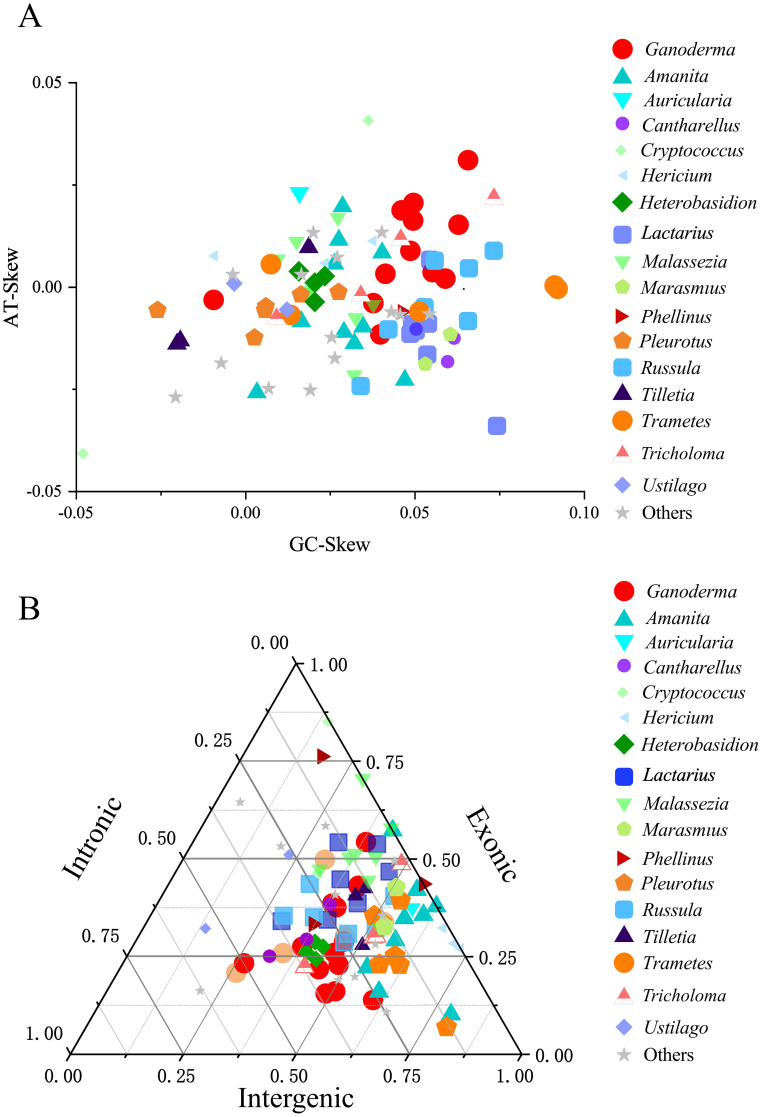
A cluster analysis of the mitochondrial genomes of the Basidiomycota phylum was conducted. Different colors and shapes represented different genera. “Others” represented a collection of genera that had only one species within the genus and had common reference value in the phylogenetic tree. **(A)** A comparison of the base offset values of the mitochondrial genomes between genera was made. **(B)** A ternary plot cluster comparison of the exon, intron, and intergenic regions of the mitochondrial genomes between genera was conducted.

**Figure 7:**
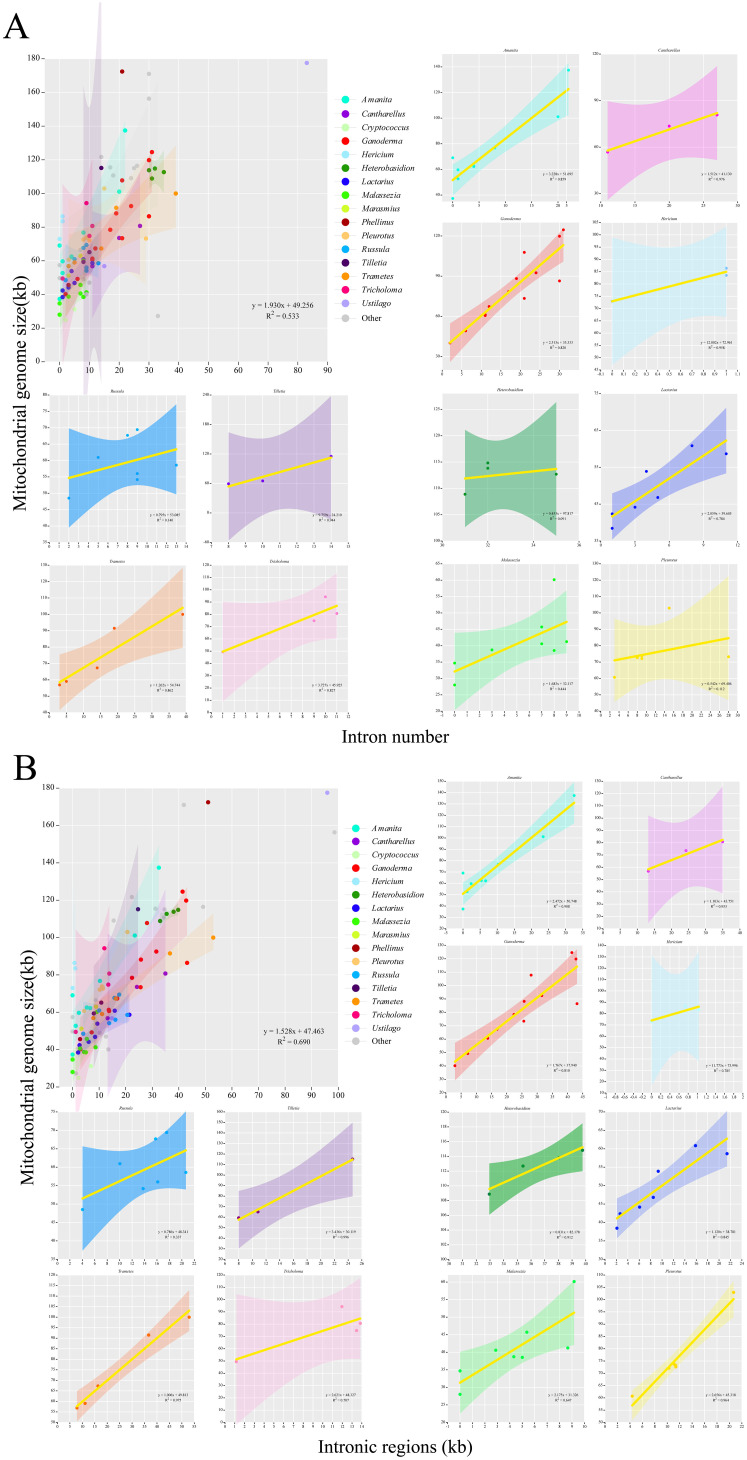
An analysis of the correlation between the length of the mitochondrial genome and the number and length of introns in the Basidiomycota phylum was conducted. Different colors represented different genera. Genera with no less than three closely related species were selected for the analysis. **(A)** An analysis of the length of the mitochondrial genome and the number of introns was conducted. **(B)** An analysis of the length of the mitochondrial genome and the length of introns was conducted.

Further visualization analysis was performed on the base bias and compositional structure of the mitochondrial genomes of the 97 Basidiomycota species. The GC-Skew and AT-Skew values ranged from negative to positive (**Figure 6A**), demonstrating diverse base composition bias among these species. Species within the same genus were generally clustered together. However, there are some exceptions. For instance, within the Cryptococcus genus, the AT-Skew ranged from -0.0407to 0.0408, and the GC-Skew ranged from -0.0480 to 0.0362. Within the Hericium genus, AT-Skew ranged from 0.0058 to 0.0113, and GC-Skew ranged from -0.0092 to 0.0377. From an interspecies perspective, we observed that species within the same genus were generally clustered together, although there were occasional deviations or multi-group clusters. For instance, in the *Ganoderma* genus, apart from *G. leucocontextum*, the species clustered together with a GC-Skew ranging from0.0377 to 0.0655 and an AT-Skew ranging from -0.0055 to 0.0311. In the *Lactarius* genus, apart from *L. volemus*, the GC-Skew ranged from 0.0486 to 0.0544, and AT-Skew ranged from -0.0165to 0.0067. Similar phenomena have been observed in the Amanita and Trametes genera. Analysis of the mitochondrial genome composition revealed significant differences (**Figure 6B**). The exon content ranged from 10. 17% to 85. 18%, the intron content varied from 0% to 63.06%, and the intergenic region content ranged from 5.38% to 79.20%. Notably, differences were observed between certain genera. For instance, closely related species within the *Cryptococcus* genus showed a large span in terms of intergenic regions (14.44%–31.53%), exonic regions (46.36%–85. 18%), and intronic regions (0.38%–22. 11%). The *Amanita*, *Phellinus*, and *Ustilago* genera also exhibit this phenomenon. However, the characteristic genera that showed differences were. Generally, within Basidiomycota, species from the same genus exhibit similar compositions. For instance, species that closely associated species within the genus have similar exon, intron, and intergenic regions. Eight genera, namely *Hericium, Heterobasidion, Lactarius*, and *Tilletia*, were clustered. They showed similar size variations in exons, introns, and intergenic regions. Cryptococcus species differed within the genus. It showed an evolutionary trend in exons, introns, and intergenic regions. Analysis was performed on the intron and mitochondrial genome lengths of 96 species within the Basidiomycota phylum (**Figure 7**). The results showed a weak correlation (R2 = 0.533) between the number of introns and length of the mitochondrial genome within the phylum. However, within 12 different genera, there was generally a strong correlation between the number of introns among species and the length of the mitochondrial genome: *Amanita* (R^2^ = 0.859), *Cantharellus*(R^2^ = 0.976), *Ganoderma* (R^2^ = 0.820), *Hericium* (R^2^ = 0.958), *Lactarius* (R^2^ = 0.784), *Tilletia*(R^2^ = 0.944), *Trametes* (R^2^ = 0.862), *Tricholoma* (R^2^ = 0.867). The remaining genera showed weak correlations: *Heterobasidion* (R^2^ = 0.091) with *Malassezia* (R^2^ = 0.444), *Pleurotus* (R^2^ = 0. 112),*Russula* (R^2^ = 0. 140) (**Figure 7A**). Among the 96 species, there was a strong correlation (R^2^ = 0.690)between intron and mitochondrial genome lengths. Within each genus, with the exception of *Russula* (R^2^ = 0.337), there was a strong correlation between intron length and mitochondrial genome length among the following species: *Amanita* (R^2^ = 0.908), *Cantharellus* (R^2^ = 0.953), *Ganoderma* (R^2^ = 0.810), *Hericium* (R^2^ = 0.785), *Heterobasidion* (R^2^ = 0.912), *Tilletia* (R^2^ = 0.996),*Trametes* (R^2^ = 0.975), *Tricholoma* (R^2^ = 0.707), *Lactarius* (R^2^ = 0.845), *Malassezia* (R^2^ = 0.647), *Pleurotus* (R^2^ = 0.964) (**Figure 7B**). Analyses were performed on 96 species of the phylum Basidiomycota. A correlation was observed between the number of introns and total mitochondrial genome length. The number of introns determines the length of the mitochondrial genome. Intron length was strongly correlated with mitochondrial genome length. This was true for both the genera and species. The results for the Ganoderma genus supported this conclusion. Intron length appeared to determine mitochondrial genome length.

### 3.6 Prediction of RNA editing sites

**Figure 8:**
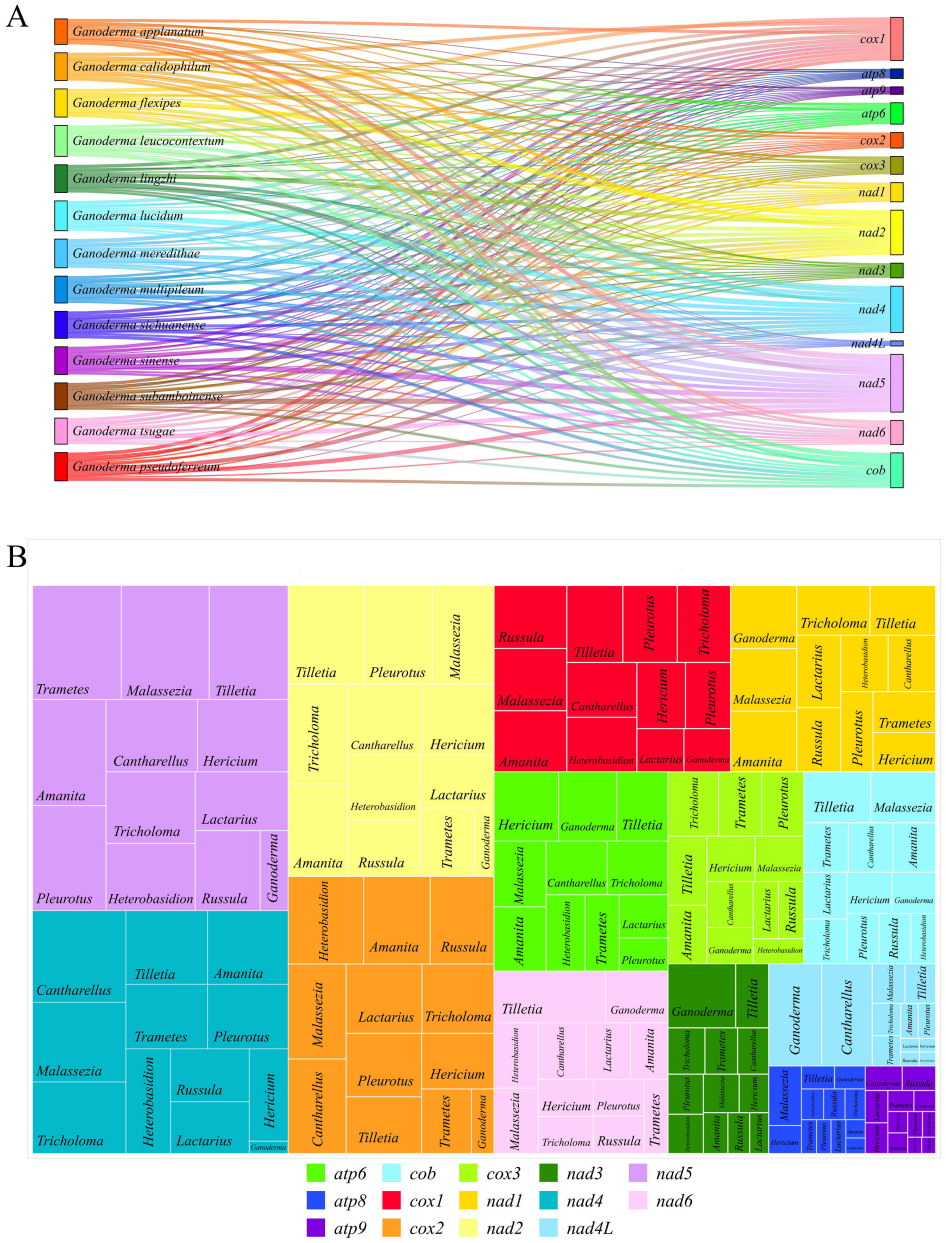
Prediction of RNA editing sites. **(A)** Sankey diagram of the number of RNA editing sites in 13 closely related species in Ganoderma. The area of the rectangle represents the number of RNA editing sites, and different colors represent different species and genes. **(B)** Distribution and proportion of RNA editing sites in 14 PCGs in 12 genera. The area of the rectangle represents the number of RNA editing sites.

Organisms regulate their genomic information through gene expression and RNA editing, thereby forming the basis of environmental adaptability. The RNA editing sites of genes reflect the number and level of specific RNA editing events, revealing the evolutionary information of the genes. (Teichert, 2020) The Deepred-MT prediction results reflected the RNA editing level of *Ganoderma* and the clusterable genera of Basidiomycota. In *Ganoderma*, the number of RNA editing sites in the 13 related species ranged from 136 to 165 (**Supplementary Table 4**). Among the 14 PCGs, *nad5* (308 RNA editing sites), *nad4* (247 RNA editing sites), *nad2* (238 RNA editing sites), and *cox1* (230 RNA editing sites) had the highest numbers of RNA editing sites, indicating a high level of RNA editing. In contrast, atp8, atp9, and nad4L had fewer editing sites, with 51, 40 and 21 RNA-editing sites (**Figure 8A**). Moreover, all predicted and qualified RNA editing sites were of the C-to-U type. The number of RNA editing sites in individual genes in the 12 genera clustered in the phylogenetic tree was predicted to test the RNA editing level of the genes. The results showed that *nad5* (364.561 per species), *nad4* (271.608 per species), *nad2* (262.277 per species), and *cox2* (249.688) had a higher number of RNA editing sites, all exceeding 200 per species and ranking among the top 14 PCGs (**Supplementary Table 5**, **Figure 8B**). The rectangular tree diagram displays the average RNA editing level of each gene among the genera. In genes with high levels of RNA editing, such as *nad5, nad4, nad2* and *cox1*, *Ganoderma* had a far lower number of RNA editing sites than the other 11 genera. Genes with a low level of RNA editing, such as *atp8, nad3, nad4L*, *Ganoderma* showed a higher number of RNA editing sites than the other 11 genera. In addition, *Russula* did not have a single qualified RNA editing site on atp6, indicating a strong conservation. All predicted and qualified RNA editing sites were of the C-to-U type (**Supplementary Table 5**, **Supplementary Figure 4**, **Figure 8B**).

### 3.7 Codon usage preference

**Figure 9:**
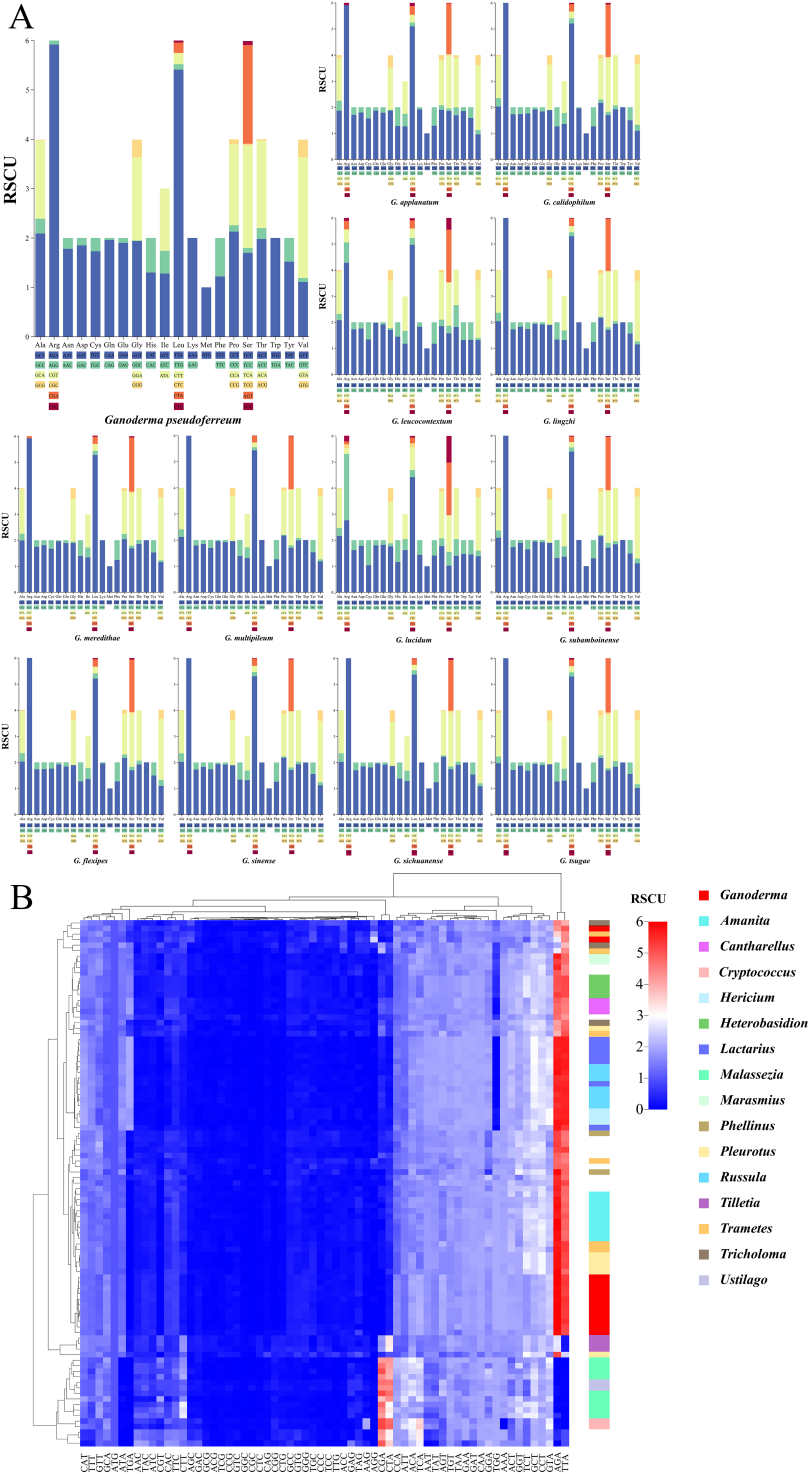
Codon usage bias analysis **(A)** Stacked bar chart of the Relative Synonymous Codon Usage (RSCU)for closely related species within the Ganoderma. **(B)** Heatmap of RSCU for 97 fungal species within the Basidiomycota, clustered using Euclidean distance. Different colors represent distinct fungal genera, and color bands indicate clustering positions at the genus level.

Visual analysis of codon usage of the 14 PCGs was conducted. The codon usage preferences of 13 closely related species of the *Ganoderma* genus were analyzed using the relative synonymous codon usage (RSCU) as the unit (**Figure 9A**). Arginine (Arg), leucine (Leu), and serine (Ser) were the most abundant amino acids. Each gene had six synonymous codons. Not all synonymous codons were present in the 13 closely related species of the *Ganoderma* genus. Their compositions were similar when Leu was encoded by these genes. *G. lucidum* has a specific codon, CTC, that is not found in other closely associated species. All synonymous codons of (Arg) were used for G. lucidum. Other closely related species predominantly use the AGA codon for Arg. In *G. leucocontextum*, with the exception of CGC, all synonymous codons are utilized. *G. applanatum, G.meredithae*, and *G. pseudoferreum* each have a unique synonymous codon for Arg, in addition to AGA. Although the remaining amino acids had distinct synonymous codon usage frequencies, these synonymous codons were conserved. This indicated the general conservation of codon usage in the mitochondrial genome within the *Ganoderma* genus. However, some closely associated species exhibit unique evolutionary trends in amino acid coding.

Further analysis was performed on the codons of 97 fungi from the Basidiomycota phylum (**Figure 9B**). Interestingly, the specific codons AGA (encoding Arg) and TTA (encoding Leu), which are most frequently used in the Ganoderma genus, were also the most commonly used in the Basidiomycota phylum. However, there are some exceptions. According to the Euclidean distance clustering results, specific codons AGA (encoding Arg) and TTA (encoding Leu) in *Malassezia, Ustilago, Cryptococcus*, and others had RSCU values less than 3, generally ranging between 0 and 1. Additional findings showed a preference for using CGA to encode arginine (Arg) and CTA to encode leucine (Leu) in these genera. Clustering results revealed that two closely related species within the Ganoderma genus were dispersed. The remaining species were grouped together, consistent with the intra-genus analysis. In general, closely associated species within the same genus are clustered together.

### 3.8 Evolutionary characteristics of gene adaptation

**Figure 10:**
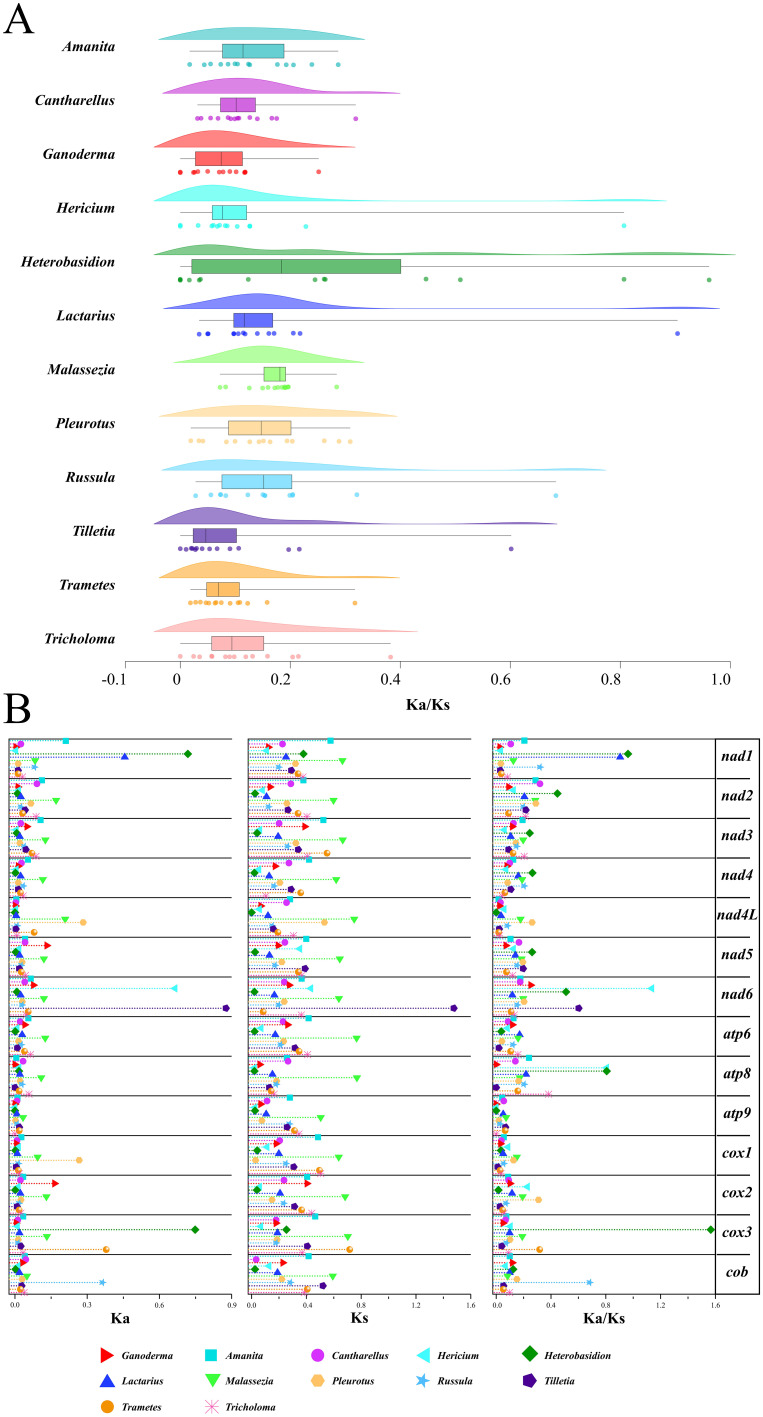
Analysis of gene replacement in 14 PCGs of Basidiomycota. **(A)** Cloud-rain diagram of the specific distribution of Ka/Ks in specific genera. The horizontal axis represents the size of the Ka/Ks value, and the vertical axis from top to bottom is: cloud diagram, representing density distribution. Box plot, representing data dispersion. Data raindrop diagram, explaining the specific size of the data. **(B)** Distribution of Ka, Ks, Ks/Ks values for each of the 14 PCGs. Different color shapes represent different genera. The horizontal axis represents the size of the value.

The Ka/Ks ratio, a measure of non-synonymous to synonymous substitution rates, indicates gene-selective pressure. A total of 14 PCGs from 12 genera were identified. Each genus was represented in at least three samples (**Figure 10A**). The *Heterobasidion* genus had the highest average Ka/Ks ratio (0.376). The Ganoderma genus had the lowest average Ka/Ks ratio (0.077). According to the box plot results (**Figure 10A**), the Ka/Ks of the Malassezia genus was the most concentrated. It also had the highest box line value. After excluding discrete data factors, the average level of Ka/Ks in the Malassezia genus was actually the highest (0. 181). The *Tilletiagenus* had the smallest Ka/Ks box line value. After removing the discrete data, the average Ka/Ks level in *Tilletia* was the lowest (0.046). The Ka/Ks cloud-rain plot showed a multipeak pattern for *Hericium*, *Heterobasidion*, *Lactarius*, *Russula*, and *Tilletia* (**Figure 10A**). This suggests negative selection evolution in at least one PCG in these genera. Analysis of the Ka/Ks ratios revealed that several protein-coding genes (PCGs) within specific genera exhibited moderate evolutionary divergence (Ka/Ks > 0.2), indicating potential adaptive variation. In Amanita, genes such as *nad1, nad2*, and *atp8* displayed elevated Ka/Ks values. In *Heterobasidion*, this trend was observed across *nad1* to *nad6* (excluding *nad4L*), as well as atp8. Similarly, *Pleurotus* showed variation in *nad2*, *nad4L*, and *nad6*; *Russula* in *nad1*, *nad4*, and *atp6*; and *Tricholoma* in *nad2, nad3, and atp8*. These findings underscore significant evolutionary differences among PCGs within these genera, suggesting lineage-specific selective pressures and functional divergence.

A thorough visual examination of the specific Ka, Ks, and Ka/Ks values of the 14 PCGs was performed (**Figure 10B**). From the graphical results of the Ka of the 14 PCGs from the 12 genera, it was observed that the Ka values of the majority of the genera (85.7%) ranged from 0 to 0. 1. This indicates that non-synonymous substitutions were not significant. In *Malassezia*, non-synonymous substitutions have been observed in 12 of 14 protein-coding genes (PCGs). Notably, *atp9* and *cob* were exceptions in which such substitutions were not detected. Significant non-synonymous substitutions were shown as Ka values greater than 0.3 in the genes nad1, nad6, and cox3 for certain species. For the 14 PCGs across the 12 species, the Ks values generally ranged from 0.2and 0.4. This suggests that synonymous substitutions are relatively common among these species.

Among these, the Ks values for Malassezia were generally higher than those of the other genera. This indicates that synonymous substitutions are more prevalent in the genus Malassezia. Visualization of Ka/Ks values from the 14 PCGs revealed two instances of Ka/Ks > 1. These genes were discovered in nad6 of Hericium and *cox3* of *Heterobasidium*. This indicates positive selection evolution. This suggests that non-synonymous mutations that lead to functional changes are more adaptive to the environment. For the remaining genes, Ka/Ks was significantly less than1. This indicates negative selection evolution. This suggests the absence of functional mutations in PCGs. Interestingly, the Ka values for atp8 and atp9 in the *Ganoderma* genus were 0. This demonstrated that no non-synonymous substitutions occurred. The Ka values for *nad5* and *cox2* in the *Ganoderma* genus were the highest among the corresponding genera but did not exceed 0.2. In addition, the Ka, Ks, and Ka/Ks values for the *Ganoderma* genus were similar to those of other genera. This suggests that the *Ganoderma* genus evolved to a certain extent within Basidiomycota and that its rate of evolution is similar to that of other genera within Basidiomycota. In contrast, the *Malassezia* genus showed notable evolution in Ka and Ks among the 14 PCGs. The *nad6* from the *Tilletia* genus had the highest Ka and Ks values. This indicates a significant difference from *nad6* of other closely related genera. This may have contributed to different ecological adaptations.

## 4 Discussion

Most fungi reproduce sexually. The nuclear genome contains parental information. This makes nuclear genome analyses complex and diverse. In most fungi, mitochondrial inheritance is uniparental, often maternal, which simplifies phylogenetic analysis. Researchers have extensively used mitochondrial genome analysis to study fungal evolution and phylogeny. The study of the fungal mitochondrial genome characteristics has become a popular research topic. Significant progress was made in the study of the characteristics and evolutionary degree of fungal mitochondrial genomes (Chen et al., 2012; Cameron, 2014; Wang et al., 2016a, b; Li et al., 2018, 2019b, 2022) In published mitochondrial genomes, closely related species of the *Ganoderma* genus are generally saprophytic fungi. Research on the mitochondrial genome characteristics of hemibiotrophic plant pathogenic fungi of the *Ganoderma* genus is scarce. However, the potential correlation between pathogenicity and mitochondrial genomic characteristics remains unclear.

In this study, we first sampled and sequenced *G. pseudoferreum* isolated from rubber tree red root disease. The mt-genome of *G. pseudoferreum* was assembled and annotated. Based on the published mitochondrial genomes of 12 closely related species in the *Ganoderma* genus, the differences and connections between the mitochondrial genomes of *G. pseudoferreum* and other12 closely related species were analyzed. *G. pseudoferreum*, with a complete genome length of 40,719 bp, is the smallest of the 13 Ganoderma species. Other closely related species had mitochondrial genomes larger than *G. pseudoferreum* by 1.2 times (*G. lingzhi*, 49,234) to 3.6 times (*G. calidophilum*, 124,588 bp). *G. pseudoferreum* has only 2 in *cox1*. Except for *G. lingzhi*, similar to *G. pseudoferreum* with 6 introns, the remaining species had at least 11 introns, with the maximum reaching 31 introns in *G. calidophilum*. Excluding the 14 shared PCGs, *G. pseudoferreum* had the lowest number of tRNAs and non-standard coding genes. Similarly, there were some differences in GC/AT-Skew compared with other closely related species. Despite differences in genome size, GC content, gene number, and GC/AT-skew, (Basse, 2010; Deng et al., 2018; Wu et al., 2021; Li et al., 2022) the key features of the mitochondrial genome remain conserved in terms of collinearity and gene displacement.

The ternary plot of mitochondrial genome component proportions revealed that introns generally constituted 50%–80% of the total within Basidiomycota. Compared with the number of introns in the mitochondrial genome, the length of introns demonstrated greater universality and reliability in determining the overall length of the mitochondrial genome. Intron regions in mitochondria typically contain structural domains of the LAGLIDADG and GIY-YIG families. Both fell under the category of Homing Endonuclease Genes (HEGs). The cyclic mechanism of HEGs is directly linked to intron loss events in mitochondrial genomes. (Goddard and Burt, 1999; Burt and Koufopanou, 2004; Bian et al., 2019) The accumulation of LAGLIDADG and GIY-YIG family structural domains in the intron regions increases the likelihood of significant intron content changes in their mitochondrial genomes. *G. pseudoferreum*, with only two introns on cox1, each housed one LAGLIDADG family open reading frame (ORFs). *G.lingzhi*, similar to *G. pseudoferreum* in mitochondrial genome size and structure, has two Group I intron ribonuclease structure domains and two LAGLIDADG family structure domains across the four introns of cox1. In the remaining closely related species of the *Ganoderma* genus, the total quantities of LAGLIDADG and GIY-YIG family structures and group I intron ribonuclease structure domains in cox1 intron regions were multiples of those in *G. pseudoferreum* and *G.lingzhi*. This likely results in widespread intron insertion/loss events in the cox1 region of *Ganoderma*. As depicted in the IPSs dynamic map, intron insertions in the *cox1* region significantly outnumbered those in other PCGs regions.

In terms of mitochondrial genome gene structure and size, *G. pseudoferreum* was similar to *G. lingzhi*. Combined with the results of the phylogenetic tree, *G. pseudoferreum* is closer to *G. lingzhi* in terms of kinship. This study used IPSs maps to study intron insertion positions in 13 closely related species of *Ganoderma*. Most introns were located in the *cox1*, *nad5*, and *cob* sections. *G. pseudoferreum* has experienced a large number of intron-loss events. *G. lingzhi*, with the highest similarity and kinship, was used to observe the order of the loss phenomena. The intron loss/insertion events on cob in *Ganoderma* occurred earlier than those in other PCGs. Intron loss/insertion events may occur in the early stages of cox1 and nad5. In the remaining 11 closely related species, IPSs maps showed some degree of intron loss or insertion. However, these differences were not as significant as those observed for *G. pseudoferreum* and *G. lingzhi*. Interestingly, *G. lingzhi* likely underwent simultaneous loss of introns in crucial genes, leading to a reduction in the mitochondrial genome size. The overall trend was towards the gene structure of *G. pseudoferreum*. Alternatively, the opposite could have occurred. Regardless of the evolutionary direction, *G. pseudoferreum* and *G.lingzhi* exhibited notable similarities in their mitochondrial genome features. In summary, intron loss within key genes of the *G. lingzhi* mitochondrial genome likely resulted in genome size reduction and structural convergence towards *G. pseudoferreum*. The significant similarity in mitochondrial genome features between the two species, combined with analyses of their phylogenetic relationship and divergence times, suggests a deep evolutionary connection.

A key distinction is pathogenicity: *G. lingzhi* is a saprophytic fungus lacking pathogenicity, whereas *G. pseudoferreum* is a root pathogen of rubber trees. The acquisition of this unique pathogenicity in *G. pseudoferreum* may stem from large-scale genomic recombination or intron editing events during environmental adaptation (e.g., host selection) within the *Ganoderma* genus. These events potentially caused extensive mitochondrial intron loss in *G. pseudoferreum*. Under directional selection, these lost introns may have been transferred to the nuclear genome, functionalized into novel genes, and ultimately conferred the ability to infect rubber trees.

The high similarity in mitochondrial structure and close phylogenetic relationship between *G. lingzhi* and *G. pseudoferreum* indicate that *G. lingzhi* may have undergone analogous intron editing and transfer processes. However, unlike *G. pseudoferreum, G. lingzhi* did not acquire specific pathogenicity. Instead, persistent mitochondrial intron editing may have driven its subsequent divergence, leading to the evolution of closely related species like *G. lucidum* and *G. sichuanense.* This hypothesis provides a genomic evolutionary framework explaining the phylogenetic relationships among *G. pseudoferreum, G. lingzhi, G. lucidum*, and *G. sichuanense*, as well as the distinct pathogenicity of *G. pseudoferreum*. It also directs research towards understanding the molecular origins of this pathogenicity. Crucially, the core mechanism – intron loss, transfer, nuclear genome integration, and functionalization – requires validation through comparative nuclear genome analysis of *G. pseudoferreum* and *G. lingzhi*.

Phylogenetic analysis based on the mitochondrial genomes of representative genera within the Basidiomycota revealed a prevalent conservation of mitochondrial genome architecture among closely related taxa. This conservation is manifested in significant similarities in intron distribution, exon composition, and intergenic spacer regions. This evolutionary pattern is particularly pronounced within the genus Ganoderma: the phylogenetic topology (**Figure 5**) exhibits a high degree of concordance with clustering based on mitochondrial genome size and compositional differences. Specifically, closely related species with smaller mitochondrial genomes (*G. pseudoferreum, G. lingzhi, G. lucidum, G. sichuanense*) exhibited homogeneity in key features such as GC content, gene number, and GC/AT skew values. This suggests a recent concerted genomic reduction event within this clade.

Codon usage preference indicates a bias towards genes with high expression levels. This further highlights the species specificity during protein translation, providing evidence for species- specific analyses and the degree of evolutionary adaptation. (Stoletzki and Eyre-Walker, 2007; Zaccaron and Stergiopoulos, 2021) The codon usage preference results for the PCGs in the Basidiomycota mitochondrial genome exhibited specificity based on Euclidean distance clustering. Despite the general preference in Basidiomycota for using the Arg-specific codon AGA and the Leu-specific codon TTA at the highest frequencies, genera with closer phylogenetic relationships on the same branch of the phylogenetic tree tended to favor CGA for encoding Arg and CTA for encoding Leu. In the context where various fungi are generally enriched with the leucine-specific codon TTA (Li et al., 2019b, 2022; Ji et al., 2023) these species (*Malasszia, Ustilago, Tilletia, Cryptococcus*) have demonstrated significant specificity. This indicates natural selection acting on these fungi, with habitat-specific pressures reshaping codon usage for translational optimization. Within closely related *Ganoderma* species, the synonymous codon repertoire for arginine (Arg) is typically limited to 1–2 options (excluding *G. lucidum* and *G. leucocontextum*). This constrained diversity suggests strong purifying selection maintaining specific codon preferences. In contrast, *G. lucidum* and *G. leucocontextum* exhibit expanded Arg codon usage, potentially reflecting adaptive evolution to enhance tRNA conversion efficiency under specific environmental constraints. Individual synonymous codons for certain amino acids in the *Ganoderma* genus showed a degree of specificity. However, the types and frequencies of synonymous codons used to encode amino acids are generally conserved.

The Ka/Ks ratio, a key metric for assessing selective pressure on genes (Teichert, 2020)(where Ka/Ks >1 suggests positive selection, <1 suggests purifying selection, and ≈1 suggests neutral evolution), was employed to analyze the evolutionary patterns of 14 core protein-coding genes (PCGs) across 12 representative Basidiomycota genera (each with ≥3 samples) (**Figure 9A**). Statistical analysis revealed significant differences in the overall distribution of Ka/Ks values for PCGs among genera. *Heterobasidion* exhibited the highest mean Ka/Ks (0.376), while Ganoderma displayed the lowest (0.077). Further analysis using box plots, after excluding the influence of outliers, indicated that Malassezia had the highest average Ka/Ks (0.181), with *Tilletia* showing the lowest (0.046). These differences demonstrate significant divergence in the intensity of selective pressure experienced by different fungal genera during evolution. Regarding the distribution patterns of Ka/Ks across PCGs, two main types were observed. The first was a clustered (peaked) distribution, exemplified by *Ganoderma*. Here, the Ka/Ks values for the majority of PCGs were tightly clustered with a pronounced peak (mean 0.077), strongly indicating pervasive and intense purifying selection across this genus, resulting in highly conserved gene sequences. The sole exception was the nad6 gene, which showed a relatively higher Ka/Ks value (an outlier point). Crucially, however, even this outlier value remained only at the average level within the broader context of Ka/Ks across the entire Basidiomycota phylum. This strongly suggests that the scale of non-synonymous substitutions within *Ganoderma* is small, not leading to significant functional changes in proteins, and its overall evolutionary pattern remains firmly within the conservative (purifying selection) realm typical of Basidiomycota PCGs. The second pattern was a relatively uniform and broad distribution, observed in the genera *Amanita*, *Heterobasidion*, *Pleurotus, Russula, and Tricholoma* (**Figure 9A**). This pattern holds significant statistical meaning, indicating substantial heterogeneity in the selective pressures acting on different PCGs within these genera. Certain genes may experience strong constraint (low Ka/Ks), while others are under relatively relaxed selection (higher Ka/Ks), reflecting functional diversification or distinct evolutionary trajectories.

Raincloud plot analysis further revealed specific evolutionary signals. The Ka/Ks distributions for *Hericium, Heterobasidion, Lactarius, Russula*, and *Tilletia* exhibited multi-modal characteristics. These results indicate that within each of these genera, at least one PCG has undergone intense purifying selection, evidenced by a Ka/Ks value significantly lower than those of other genes in the genus, forming a distinct, separate “peak”. The instances of strong purifying selection identified on individual PCGs in Hericium and *Heterobasidion* (**Figure 9A**), in particular, highlight lineage-specific evolutionary trajectories. This suggests these genera may have undergone adaptive evolution related to specific gene functions or experienced unique evolutionary bottlenecks, representing potential novel trends in their evolutionary direction.

RNA editing is a mechanism by which organisms can flexibly diversify their protein conformations and functions to adapt to the environment and promote biological evolution through insertion, deletion, and modification events. (Xin et al., 2023) In fungi, RNA editing typically involves the conversion of the specific editing site, cytosine(C), to uracil(U), under the action of cytidine dehydrogenase (Zaccaron and Stergiopoulos, 2021)Additionally, adenine-to-inosine (A-to-I) RNA editing occurs under the action of adenosine dehydrogenase, which is common in animal cells, but rare in fungi. However, existing research has demonstrated that A-to-I RNA editing, a fungus-specific editing mode, provides fungi with important adaptability to sexual reproduction. (Xin et al., 2023) Based on phylogenetic clustering analysis within the Basidiomycota, we observed a significant inverse correlation between the level of RNA editing (i.e., the number of predicted editing sites) and the evolutionary conservation of protein-coding genes (PCGs). The prevailing pattern indicates that highly conserved genes typically exhibit lower levels of RNA editing. For instance, genes such as *atp8, atp9, nad3*, and *nad4L* display average editing sites concentrated between 1 and 6 (generally not exceeding 10) across most genera, reflecting their high degree of conservation. Conversely, genes with higher levels of RNA editing, such as *atp6, cob, cox1, nad1, nad2, nad4*, and *nad5*, generally exhibit lower conservation. While this correlation holds true for the majority of genera, notable exceptions demonstrating atypical conservation patterns exist. These include certain genes within *Ganoderma*, *Cantharellus*, and *Malassezia* showing unexpectedly low conservation, and specific genes in *Lactarius, Pleurotus*, and *Russula* (most strikingly, *atp6* in *Russula* with 0 editing sites) exhibiting atypically high conservation.

Particularly striking is the distinct pattern observed in the genus *Ganoderma*, which markedly contrasts with the other 11 genera analyzed. In genes typically associated with high editing levels and thus lower conservation (*nad5, nad4, nad2, cox1*), the inter-generic average number of editing sites in *Ganoderma* was significantly lower than in the other genera, implying these genes are, conversely, highly conserved in Ganoderma. Conversely, in genes typically associated with low editing levels and high conservation (*atp8, nad3, nad4L*), the number of editing sites in *Ganoderma* was higher than in the other genera, indicating comparatively lower conservation for these genes. This inverse relationship between gene conservation and RNA editing levels strongly suggests that *Ganoderma* has adopted a divergent, or even opposing, molecular evolutionary strategy in adapting to environmental pressures compared to its closely related genera within the Basidiomycota. At the phylum level, the phylogenetic tree clearly demonstrates monophyly at the family level, with all species belonging to the same family clustering within distinct branches. Notably, the phylogenetic topology reconstructed from the concatenated dataset of 14 protein-coding genes (PCGs) showed high congruence with previously published frameworks for Basidiomycota phylogeny, indicating that mitochondrial PCGs harbor robust phylogenetic signal. Within the genus Ganoderma, nodes reconstructed from the mitochondrial genome data with high statistical support robustly supported the taxonomic delineation of species. Collectively, these results confirm that the mitochondrial genome serves as an effective molecular marker for resolving phylogenetic relationships among Basidiomycota fungi, and its evolutionary dynamics provide crucial evidence for understanding the history of lineage diversification (Wang et al., 2012; Kuok et al., 2013; Chien et al., 2015).

Fungi of the *Ganoderma* genus are characterized by their widespread global distribution and diverse features. (Kües et al., 2015; Kwon et al., 2016; Jargalmaa et al., 2017) However, the identification of fungi within the Ganoderma genus presents certain challenges. (Wang et al., 2012; Li et al., 2022) Owing to their inherent advantages, mitochondrial genomes are widely utilized in studies related to molecular markers and the degree of phylogenetic evolution. (Cameron, 2014; Yuan et al., 2015; Li et al., 2019b; Ji et al., 2023) *G. pseudoferreum*, a rare plant pathogen of the Ganoderma genus, have not yet been reported. It remains unknown whether the mitochondrial genome of *G. pseudoferreum*, a plant pathogen, has undergone changes compared to that of common saprophytic fungi in the Ganoderma genus. In this study, we assembled the first mitochondrial genome of *G. pseudoferreum*, a pathogen that causes rubber tree red root disease. We observed differences in the composition and characteristics of the *G. pseudoferreum* mitochondrial genome by comparing it with previously published mitochondrial genomes of closely related species in the Ganoderma genus. Furthermore, we placed the Ganoderma genus within Basidiomycota for intergeneric comparisons to observe the overall evolutionary degree of the Ganoderma genus within Basidiomycota. These results reveal significant differences in the composition and structure of the mitochondrial genome of the plant pathogen *G. pseudoferreum*. The characteristics of *G. pseudoferreum* represent a new potential evolutionary direction for saprophytic fungi of the Ganoderma genus. This study provides new insights into plant pathogenic fungi in the Ganoderma genus and offers a fresh perspective on the evolutionary patterns of fungi in the Ganoderma genus. It had been demonstrated that the mitochondrial genome, as a molecular marker, is important for studying the phylogenetic and kinship relationships of the Ganoderma genus and Basidiomycota.

## Data Availability

The datasets presented in this study can be found in online repositories. The names of the repository/repositories and accession number(s) can be found below: https://www.ncbi.nlm.nih.gov/, PP778499.
