# Supplementary figures and images for: Characterization of Ganoderma pseudoferreum mitogenome revealed a remarkable evolution in genome size and composition of protein-coding genes
Source: Front Plant Sci. 2025 Aug 20;16:1532782. doi: 10.3389/fpls.2025.1532782 (PMC12405218; doi:10.3389/fpls.2025.1532782)

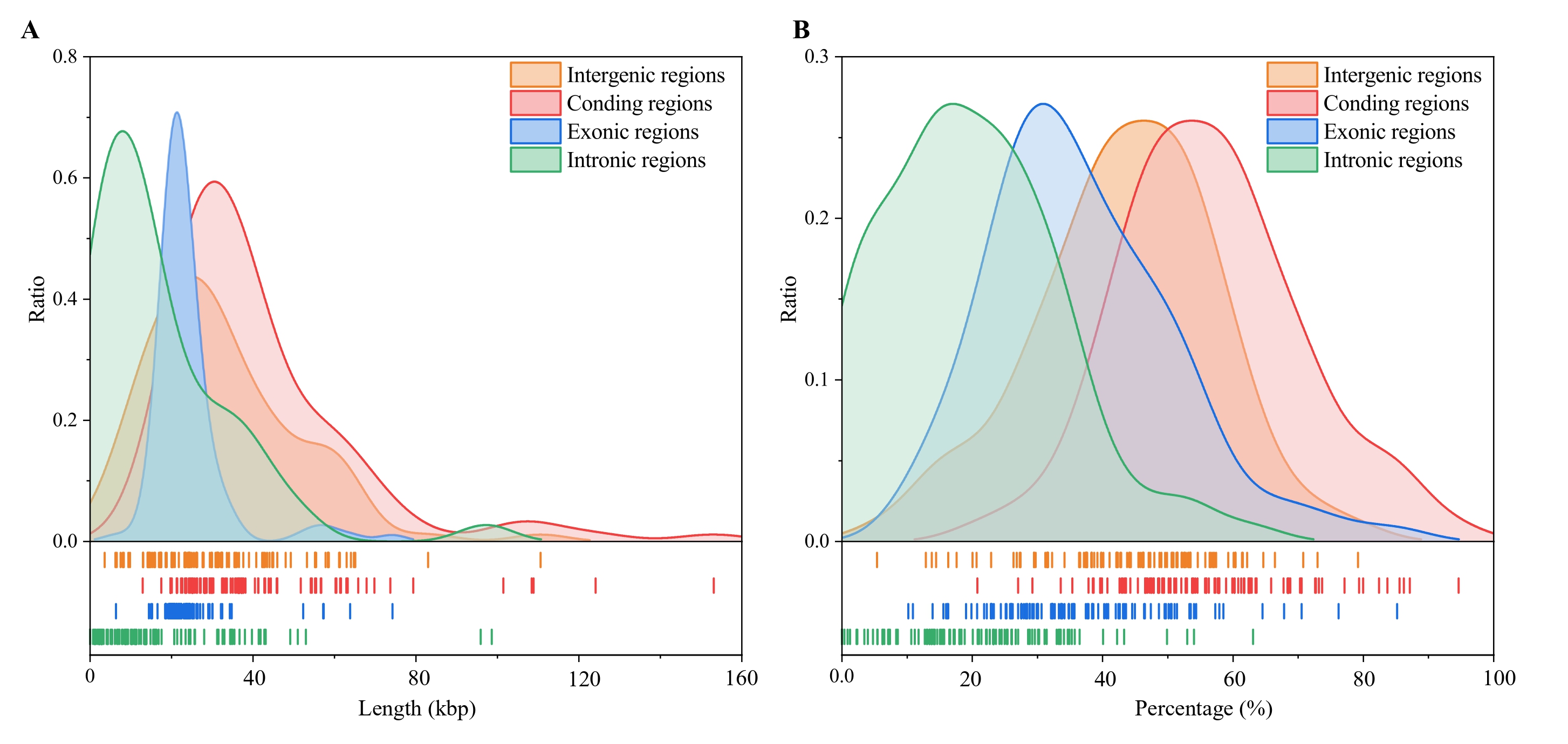

Supplement: Supplementary file 3 [file Image3.jpeg]

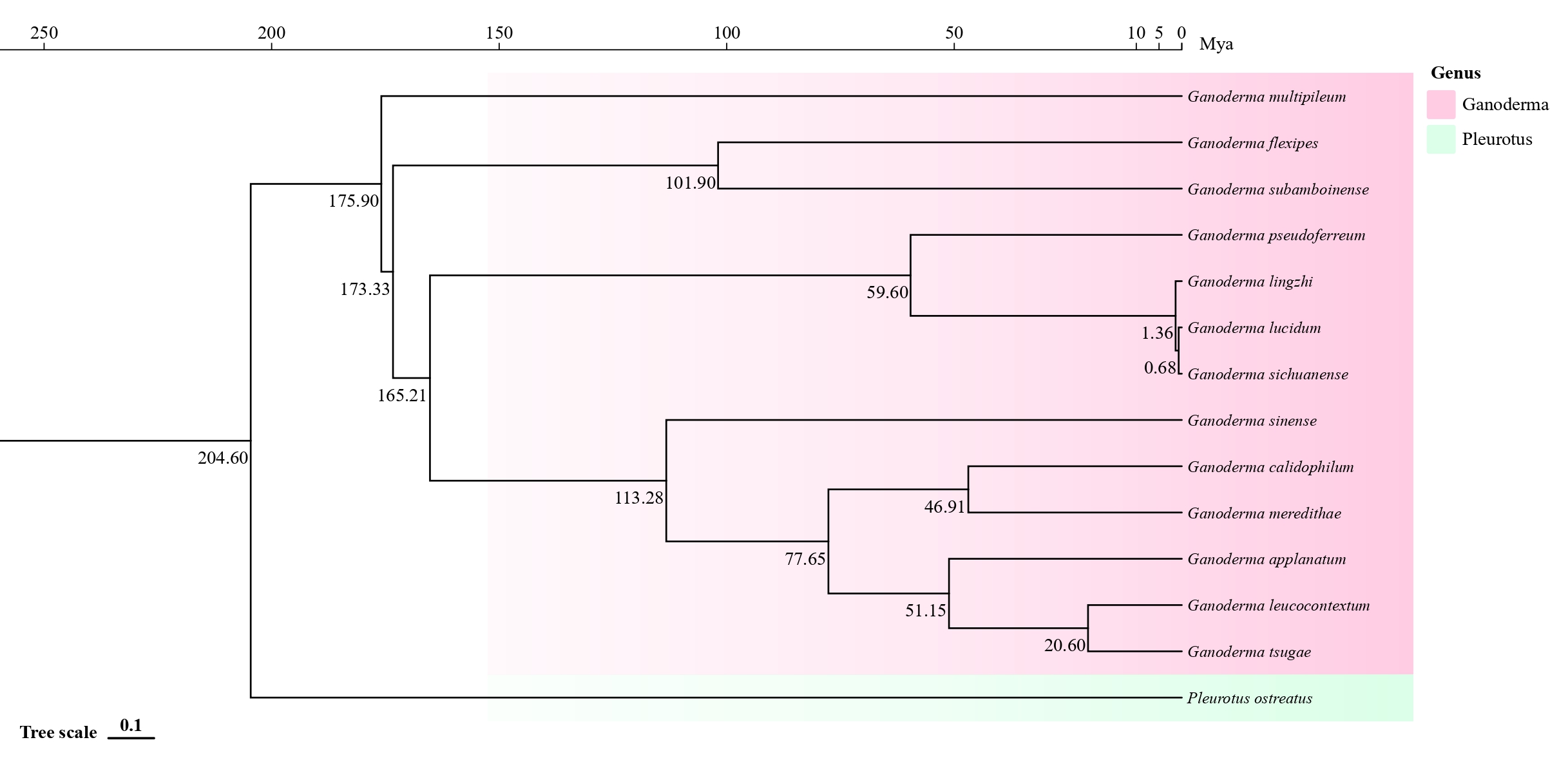

Supplement: Supplementary file 4 [file Image4.jpeg]
